# Label-free ovarian cancer histopathological diagnosis using two-photon autofluorescence microscopy with a joint denoising and segmentation framework

**DOI:** 10.1038/s41377-026-02464-6

**Published:** 2026-08-27

**Authors:** Zhengyuan Pan, Naikun Song, Shanshan Cheng, Wen Pang, Hongen Liao, Yu Wang, Bobo Gu

**Affiliations:** 1 School of Biomedical Engineering, Shanghai Jiao Tong University, Shanghai, 200030 China; 2 Department of Gynecology, Shanghai First Maternity and Infant Hospital, School of Medicine, Tongji University, Shanghai, 200092 China; 3 Shanghai Key Laboratory of Flexible Medical Robotics, Tongren Hospital, Institute of Medical Robotics, Shanghai Jiao Tong University, Shanghai, China

**Keywords:** Imaging and sensing, Biophotonics

## Abstract

Two-photon autofluorescence (TPAF) microscopy is a promising modality for rapid, label-free assessment of unstained tissue, but is fundamentally limited by the poor visibility of nuclei, which are one of the gold-standard cancer biomarkers, due to their negative contrast nature. A restoration method capable of distinguishing these signal voids from stochastic noise to render quantitative pathology is lacking. Here, we proposed a deep learning framework of AFN-DeSeg (Auto-Fluorescence Nuclei Denoising and Segmentation) to unify image denoising and nuclear segmentation via a dual-encoder architecture incorporating a DINOv3 vision transformer. By explicitly modeling detector/shot noise while optimizing for morphological fidelity, AFN-DeSeg recovered diagnostic-grade nuclear features from noisy signals. We demonstrated that AFN-DeSeg achieved a superior reconstruction fidelity compared to state-of-the-art sequential and joint denoising-segmentation pipelines and yielded a high correlation with hematoxylin and eosin (H&E) pathology in identifying key diagnostic areas of high-grade serous ovarian carcinoma. The proposed AFN-DeSeg bridges the gap between label-free TPAF imaging and H&E-based histology, establishing a computational foundation for label-free morphological pathology and toward future intraoperative optical biopsy.

**Figure:**
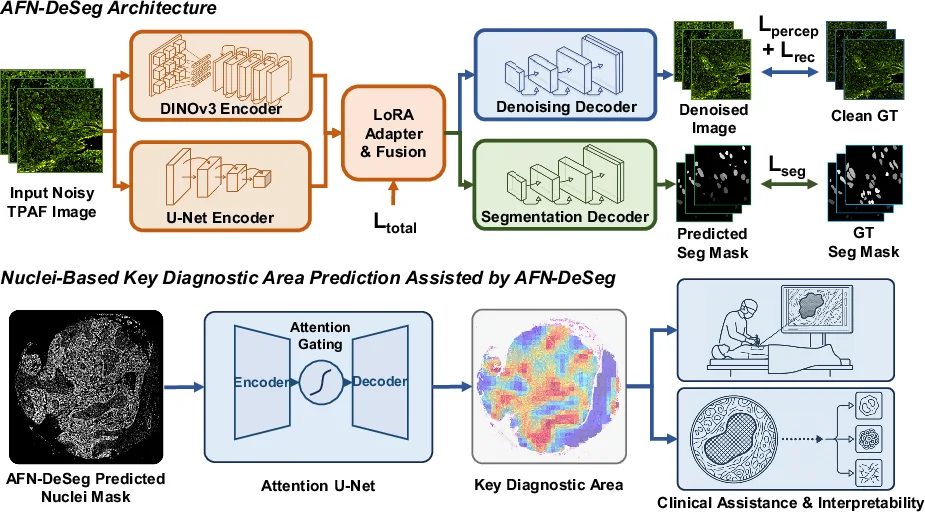

## Introduction

Ovarian cancer, particularly epithelial ovarian cancer, remains one of the most lethal gynecologic malignancies globally, frequently termed the silent killer due to its insidious onset and lack of specific early symptoms^1,2^. Despite advances in targeted therapies, a fundamental inflection point of long-term survival rates has not achieved over recent decades^3–5^. Due to the limitation of current screening strategies, over 70% of patients are diagnosed at advanced or terminal stages^6^. The current gold standard for diagnosis, Hematoxylin and Eosin (H&E) histopathology, is an ex vivo, time-consuming process unsuitable for real-time intraoperative decision-making. Surgeons often rely on intraoperative frozen section analysis, but this technique still suffers from freezing artifacts, sampling errors in heterogeneous masses, and low sensitivity for borderline tumors, leading to potential misdiagnosis and inadequate surgical management. However, there is an urgent clinical need for biopsy technologies capable of providing rapid, label-free histopathological assessment with the ultimate goal of in situ intraoperative deployment.

Among emerging optical modalities, Two-photon autofluorescence (TPAF) microscopy stands out for its deep tissue penetration, intrinsic optical sectioning, and label-free contrast from endogenous fluorophores including nicotinamide adenine dinucleotide (NADH) and flavin adenine dinucleotide (FAD)^7–9^. However, the clinical deployment of TPAF imaging is hindered by fundamental physical constraints i.e., the photon starvation regime required to prevent phototoxicity. This results in low Signal-to-Noise Ratios (SNR) and complex Mixed Poisson-Gaussian (MPG) noise for imaging the tissue with low two-photon absorption^10–12^. Furthermore, unlike stained nuclei in H&E images, nuclei in label-free TPAF images appear as signal voids i.e., negative contrast embedded in noisy cytoplasm. This coupling of high noise and ambiguous contrast compromises rich pathological information of nuclei and downstream computational analysis.

To address the above mentioned bottlenecks, deep learning has revolutionized microscope image processing. In the domain of denoising, supervised methods like DnCNN^13^ and CARE^14^ set performance benchmarks but require paired clean-noisy data, which is physically impossible to acquire in dynamic live tissue. Self-supervised approaches, such as Noise2Void (N2V)^15^, Noise2Fast^16^, and Noise2Self^17^, overcome this issue by learning from noisy data alone using blind-spot networks and down-sampling strategies, respectively. However, these methods often lack semantic awareness, leading to the over-smoothing of fine textures critical for segmentation and diagnosis. As for segmentation, the classic U-Net^18^ has been superseded by instance segmentation models that handle crowded cells better. StarDist^19^ uses star-convex geometric priors, while Cellpose^20–22^ utilizes vector flow fields to robustly separate touching cells. More recently, foundation models like the Segment Anything Model (SAM)^23^ have demonstrated impressive zero-shot capabilities. However, SAM is pre-trained on natural images, and suffers an accuracy drop when applied to medical and microscope images. MedSAM^24^, Micro-SAM^25^, Cellpose-SAM^26^, and other parameter-efficient fine-tuning methods^27,28^ have attempted to bridge this gap. Despite these advances, denoising and segmentation are typically treated as sequential tasks, the sequential denoise-then-segment workflows are prone to error propagation, where aggressive denoising destroys weak boundary cues. While joint learning frameworks like DenoiSeg^29^ and Cellpose 3 model^22^ attempt to unify these tasks, they are often limited by shallow architectures, making it unfeasible to handle the complex noise patterns like TPAF noise. Furthermore, recent advancements in computational optics continue to highlight the need for physics-aware constraints in deep learning models to ensure data fidelity^30–36^.

**Fig. 1:**
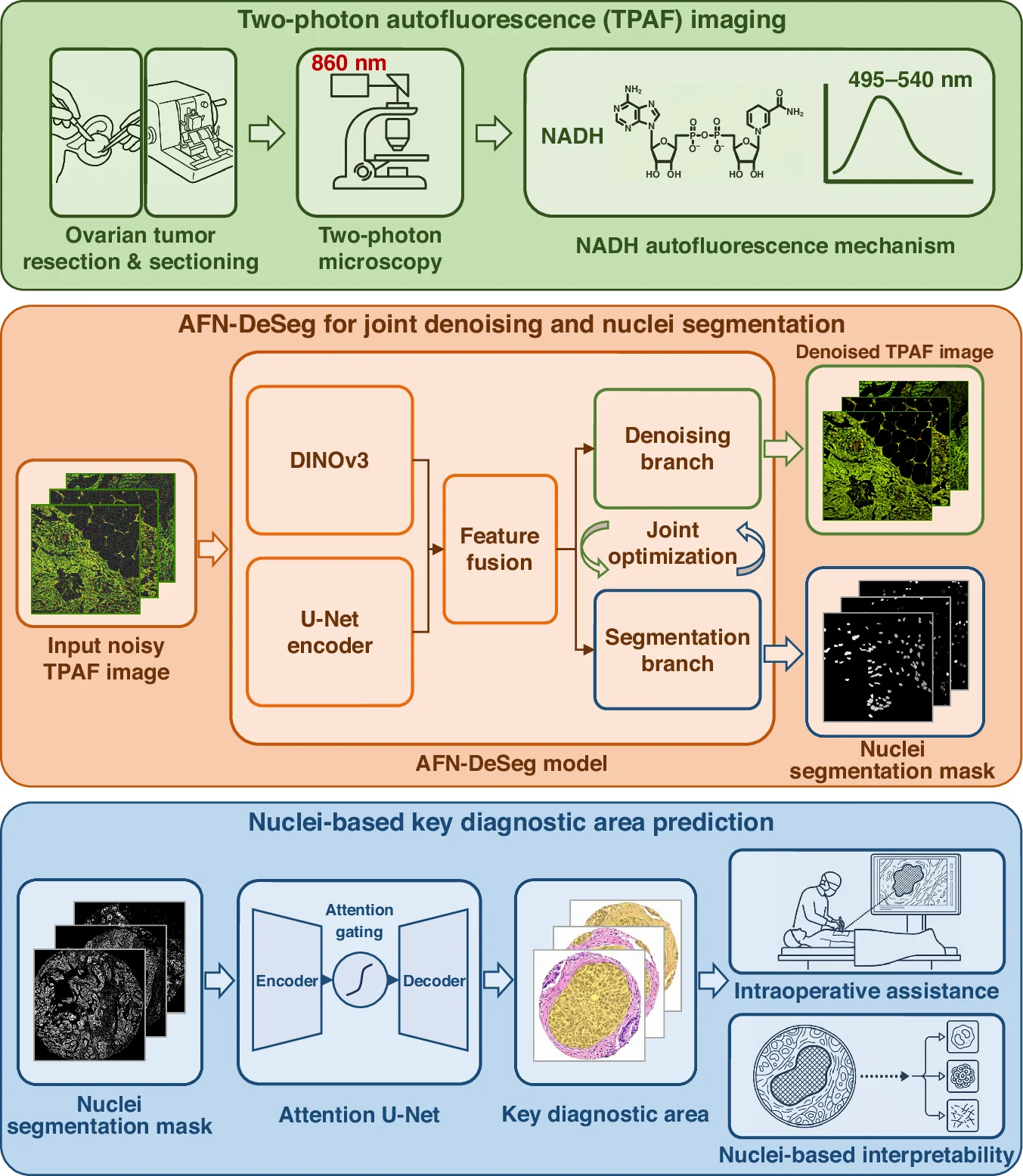
Analysis workflow of AFN-DeSeg for ovarian cancer assessment and key area identification based on label-free two-photon autofluorescence imaging on formalin-fixed and paraffin-embedded (FFPE) tissue sections

To bridge the gap between TPAF images and histopathology, we proposed and demonstrated a joint denoising and segmentation framework named AFN-DeSeg (Auto-Fluorescence Nuclei Denoising and Segmentation) for ovarian cancer diagnosis based on TPAF images (Fig. 1). The proposed framework integrates DINOv3 model^37^, a state-of-the-art vision foundation model, with Low-Rank Adaptation (LoRA)^27^ to efficiently capture the unique texture of ovarian tissue. By incorporating an instrument-calibrated MPG noise model and a dual-stream joint optimization strategy, AFN-DeSeg simultaneously recovers high-SNR TPAF images and nuclear masks. Furthermore, we demonstrate that the nuclear masks can be directly utilized to predict key pathological diagnostic areas with high concordance to H&E standards. It potentially provides interpretability for diagnostic models, computational support for label-free morphological pathology, and a foundation for future intraoperative TPAF-based optical biopsy (Fig. 1).

## Results

### AFN-DeSeg model design

**Fig. 2:**
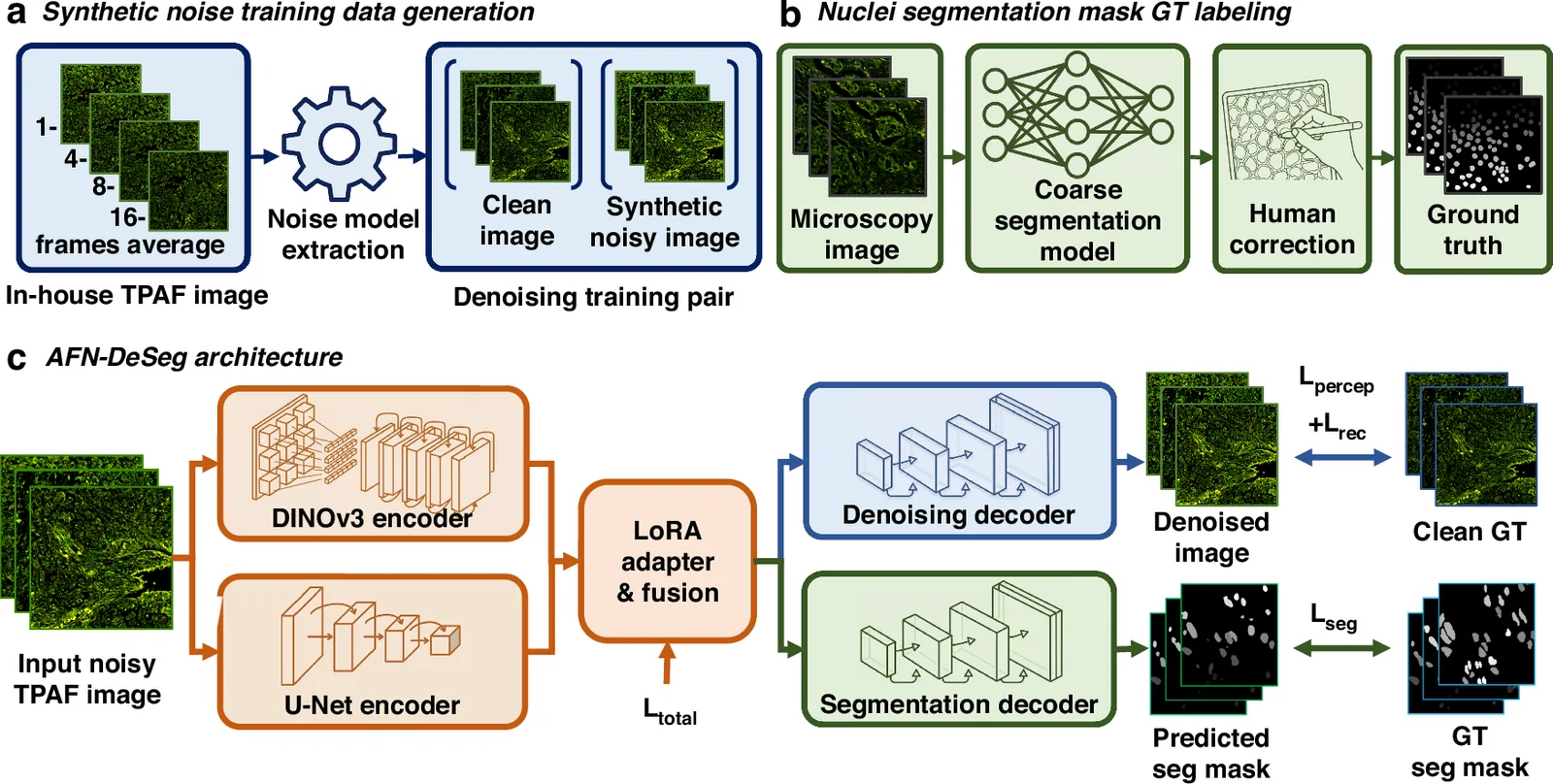
**Model of the proposed AFN-DeSeg framework**. **a** Synthetic denoising-data generation based on mixed Poisson and Gaussian noise model. **b** Human-in-the-loop nuclei mask GT labeling. **c** AFN-DeSeg architecture based on dual-stream encoder and mutual reinforcement of denoising and segmentation tasks

Standard segmentation approaches typically rely on high-contrast boundaries, but TPAF imaging presents unique challenges: negative contrast of nuclei and low signal-to-noise ratio (SNR) that obscures nuclear morphology. These challenges motivated us to construct a framework that jointly addresses denoising and negative-contrast nuclei segmentation (Fig. 2). To train a model capable of distinguishing signal from complex TPAF noise without paired experimental data, we first established a noisy data synthesis pipeline based on an instrument-calibrated MPG noise model for training the denoising branch (Fig. 2a). We acquired FOVs with different numbers of frames representing different levels of noise, and explicitly modeled the TPAF noise distribution as a mixed Poisson-Gaussian process with parameters calibrated to our specific imaging system. This instrument-calibrated synthesis enables the network to learn the statistical properties of TPAF imaging, bridging the domain gap between synthetic and real-world data.

To validate our approach, we established a robust benchmarking foundation via a human-in-the-loop annotation workflow (Fig. 2b). Recognizing the difficulty of labeling noisy TPAF data, we employed a coarse segmentation model to generate initial masks on clean reference images, which were subsequently corrected by human. This process created a high-fidelity ground truth (GT) dataset, ensuring that our downstream evaluations reflect genuine anatomical accuracy.

Building on these data, we developed the AFN-DeSeg framework (Fig. 2c), which addresses the inherent challenges of low-SNR and negative contrast through a dual-stream design. First, to resolve the ambiguity of negative-contrast nuclei, we integrated a DINOv3 Vision Transformer finetuned on microscopy data alongside a standard U-Net encoder. The multi-scale features are integrated via a LoRA Adapter & Fusion module, enabling efficient adaptation to the TPAF domain. Unlike convolutional networks that rely on local edge gradients, the DINOv3 stream leverages self-attention mechanisms to capture global features. This capability enables the model to infer the presence of ‘ghost’ nuclei and reconstruct discontinuous boundaries even in high-noise regions where local gradients fail. Furthermore, the foundation model’s semantic priors allow it to discriminate true nuclei from morphological mimics and disentangle crowded instances, ensuring robust segmentation based on biological structure.

Second, we employed a mutual reinforcement strategy between denoising and segmentation branches based on a shared encoder. Rather than training separate networks, the denoising and segmentation branches stem from the same encoder fusion block, optimizing a composite loss function (${L}_{{total}}$). This shared representation strategy leverages the complementary nature of the tasks to overcome their individual limitations. The denoising objective (${L}_{{\mathrm{rec}}}+{L}_{{\mathrm{percep}}}$) acts as a structural regularizer, forcing the encoder to capture fine-grained spatial details and high-frequency edges, which prevents the segmentation branch from overfitting to noise artifacts and ensures precise boundary delineation. Simultaneously, the segmentation objective (${L}_{{\mathrm{seg}}}$) provides a semantic prior, compelling the encoder to prioritize object-level coherence over pixel-level statistics. This prevents the denoising branch from interpreting the negative-contrast nuclei as background fluctuations to be smoothed out, ensuring that the restored images retain distinct morphological fidelity even in low-SNR regions.

### Evaluation of AFN-DeSeg denoising performance on TPAF images of ovarian cancer tissue

**Fig. 3:**
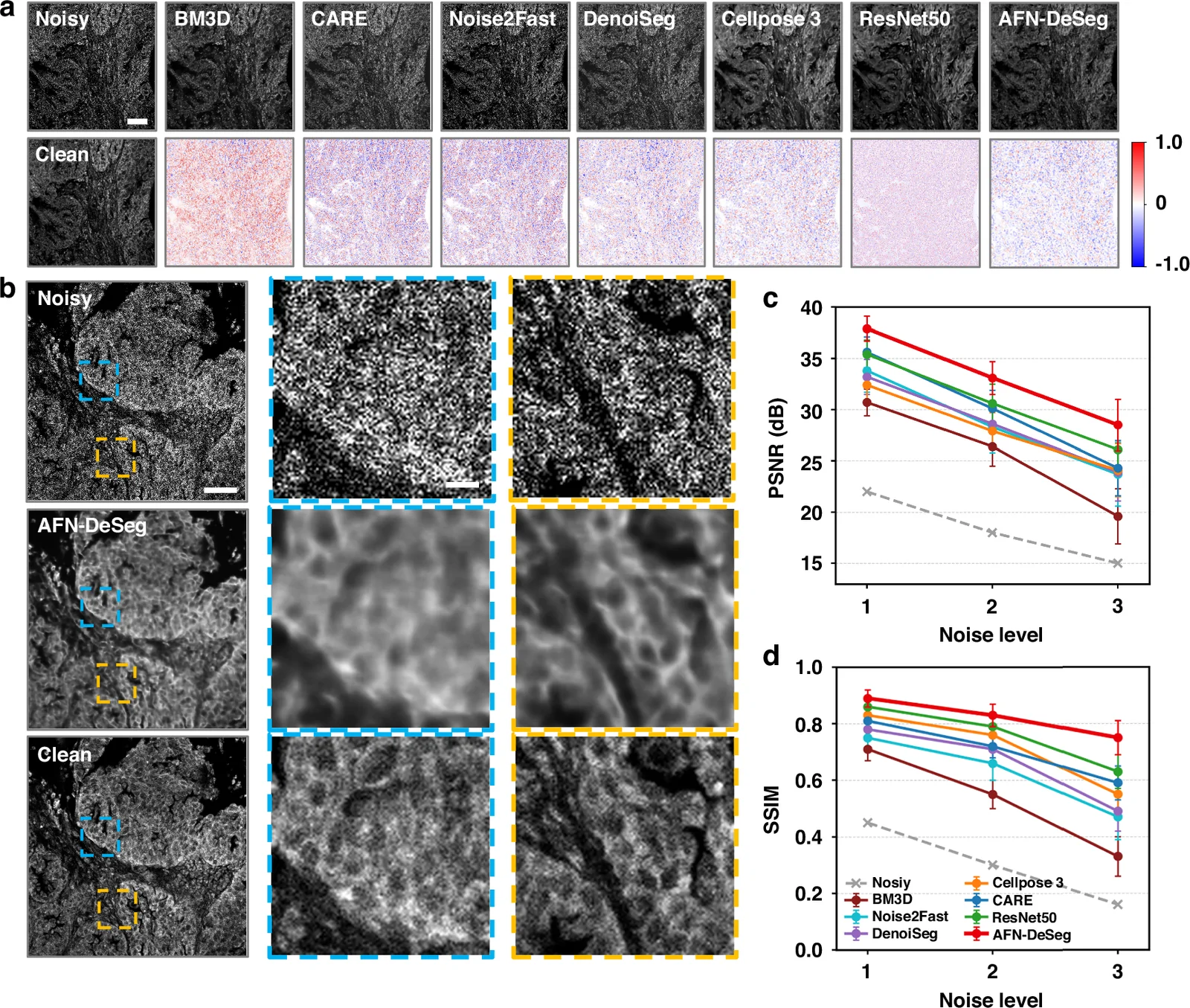
**Denoising performance on TPAF images of ovarian cancer tissue**. **a** Comparison of denoised outputs between the proposed AFN-DeSeg and other baseline methods (BM3D, CARE, Noise2Fast, DenoiSeg, Cellpose 3, and ResNet50 baseline). Color bar indicates the normalized reconstruction error. Scale bar: 50 µm. **b** Representative TPAF images illustrating noisy input, AFN-DeSeg output, and clean GT, with high-magnification insets highlighting preservation of nuclear voids and cytoplasmic texture. Main scale bar: 50 µm; zoom-in scale bar: 10 µm. **c**, **d** Quantitative evaluation of denoising fidelity using PSNR and SSIM across three noise levels (*N* = 500). All TPAF images in this figure were acquired in NADH channel (495–540 nm)

The restoration performance of AFN-DeSeg model was evaluated using TPAF images of ovarian cancer tissue affected by MPG noise. The test dataset consisted of both real noisy images and synthetic noisy-clean image pairs. The noisy image was used as input, which mimicked the low-SNR conditions of intraoperative TPAF images. The denoised image by AFN-DeSeg is compared with the ground-truth image and the denoised images by six other baseline methods, i.e., BM3D, CARE, Noise2Fast, DenoiSeg, Cellpose 3, and ResNet50 (Fig. 3a). The nuclei in the raw noisy images are signal voids obscured by strong photon shot noise and are too ambiguous to be discerned. AFN-DeSeg is capable of reducing the MPG noise artifacts while resolving the boundaries of these negative-contrast nuclei. This is also consistent with the residual maps, where AFN-DeSeg shows the lowest and most uniform error distribution of structural residuals as compared with other baselines (Fig. 3a, bottom).

To further verify the denoising capabilities of AFN-DeSeg, the nuclei morphological appearances were examined (Fig. 3b). The proposed method successfully suppresses heavy granular noise while transforming the TPAF image to a clean, smooth representation with sharply defined nuclear boundaries. Notably, AFN-DeSeg efficiently resolves the edges of signal voids and makes them discernible for human experts and segmentation algorithms. This improvement can be attributed to the synergy of the U-Net and DINOv3 encoder, jointly capturing both precise local textures and robust semantic structures.

The performance of the proposed AFN-DeSeg was further assessed by pixel-level fidelity and structural preservation, which were quantified using PSNR and SSIM, respectively (Figs. 3c, d). The attained PSNR by AFN-DeSeg consistently exceeds that of the competing methods across all noise regimes. The SSIM of the network output is also better than that of the baselines, maintaining values above 0.75 even under severe noise conditions, while other methods exhibit notable degradation. Due to its instrument-calibrated noise modeling, the restored images maintain high fidelity without extensive manual parameter tuning.

### Evaluation of AFN-DeSeg nuclei segmentation performance on TPAF images of ovarian cancer tissue

**Fig. 4:**
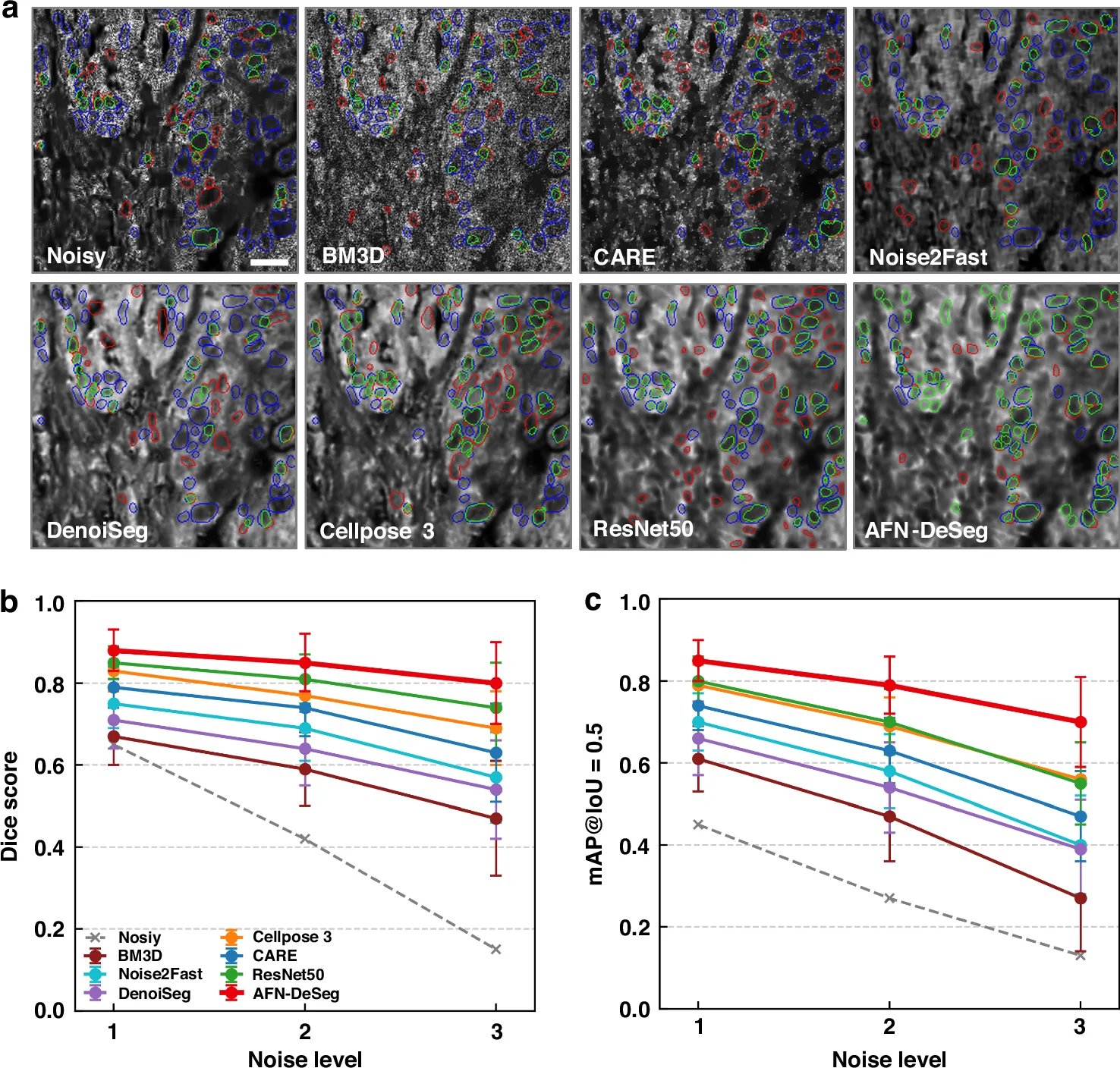
**Nuclei segmentation performance on TPAF images of ovarian cancer tissue**. **a** Representative segmentation results using baseline methods (BM3D+cyto2, CARE+cyto2, Noise2Fast+cyto2, DenoiSeg, Cellpose 3, and ResNet50) and the proposed AFN-DeSeg at three noise levels. GT nuclei mask (blue), prediction nuclei mask (red), and overlapping areas of prediction and GT (green) are overlaid on the denoised images for visual comparison. Scale bar: 20 µm. Quantitative segmentation metrics of **b** Dice Score and **c** mean Average Precision @ IoU = 0.5 across different noise conditions (*N* = 500). All TPAF images in this figure were acquired in NADH channel (495–540 nm)

The segmentation performance of the proposed AFN-DeSeg model was evaluated on TPAF images of ovarian cancer tissue (Fig. 4), where accurate nuclei delineation is intrinsically difficult due to their negative contrast of signal voids, irregularity of tumor nuclei shape, and MPG noise. AFN-DeSeg took the noisy TPAF image as input (Fig. 4a, top left), and generated an instance segmentation mask (not shown directly, but visualized as contours). The segmentation masks are compared against ground-truth masks (Fig. 4a, bottom left) and predictions from other baseline strategies including sequential denoising-segmentation (denoising-only method followed by cyto2 segmentation model), and joint denoising and segmentation methods (DenoiSeg, Cellpose 3, and ResNet50). It is observed that baseline methods struggle to distinguish the negative contrast of TPAF nuclei from background noise. Sequential denoising-segmentation workflows (BM3D, CARE, and Noise2Fast followed by cyto2 segmentation) normally misinterpret cytoplasmic speckle noise as nuclear voids, leading to over-segmentation. To rigorously evaluate performance on specific nuclei-level features, AFN-DeSeg is compared against the benchmark Cellpose 3 and H&E references (Fig. S1). While Cellpose 3 often fails to handle complex morphologies, AFN-DeSeg exhibits a high level of consistency with H&E histology and cancer pathology features. Specifically, AFN-DeSeg accurately delineates the varied shapes and sizes of tumor nuclei atypia, and efficiently separates aggregated and adjacent nuclei instances, it enables AFN-DeSeg to reliably segment vesicular chromatin and substantially reduce the false-positive segmentation of structurally identifiable non-nuclei vesicles. The global semantic context provided by the DINOv3 encoder allows the model to distinguish true biological voids from noise-induced signal dropouts or vesicles.

The segmentation performance is further quantitatively evaluated. AFN-DeSeg consistently outperforms competing methods in both global pixel-level overlap (Dice Score) and instance-level segmentation precision (mAP@IoU = 0.5) across varying noise levels (Figs. 4b, c). Moreover, AFN-DeSeg surpasses the strongest baseline of ResNet50 by ΔDice ≈ 0.06 at the highest noise level (0.80 vs 0.74 at *n* = 1), and its superiority over the from-scratch and Cellpose-based methods is further enhanced as noise increases (e.g., a Dice gap of >0.10 vs Cellpose 3 at high noise; Table S3). This indicates that AFN-DeSeg maintains robust instance separation, while baseline methods suffer from instance merging or omission in low-SNR conditions. This resilience is ascribed to the joint optimization strategy, where segmentation gradients refine the feature extractor, enabling the model to prioritize structural cues essential for delineating signal voids even when the signal-to-noise ratio is extremely low.

AFN-DeSeg substantially increases nuclear visibility, which was quantified at pixel and functional levels. AFN-DeSeg improves PSNR by 16.6 dB on average across all noise levels relative to noisy inputs (Fig. 3c) at pixel level, confirming that the denoising operation restores the underlying signal rather than merely producing a perceptually plausible image. At the functional level, nuclear visibility is operationally defined as the ability of a downstream algorithm to localize and delineate nuclei. The standardized fine-tuned cyto2 segmenter (Note S5.7) yields Dice ~0.15 at the most challenging single-frame regime (*n* = 1), whereas AFN-DeSeg reaches Dice ~0.80 (Table S3, Fig. 4b), indicating a 5.3-fold improvement in segmentability. At the easier case of *n* = 8, the corresponding Dice values reach 0.65 vs 0.88. This functional index of nuclear visibility is clinically meaningful, since downstream diagnostic tasks (nuclear counts, morphology, key-area prediction) depend directly on segmentation success.

### Comparative evaluation of AFN-DeSeg with H&E histopathology

**Fig. 5:**
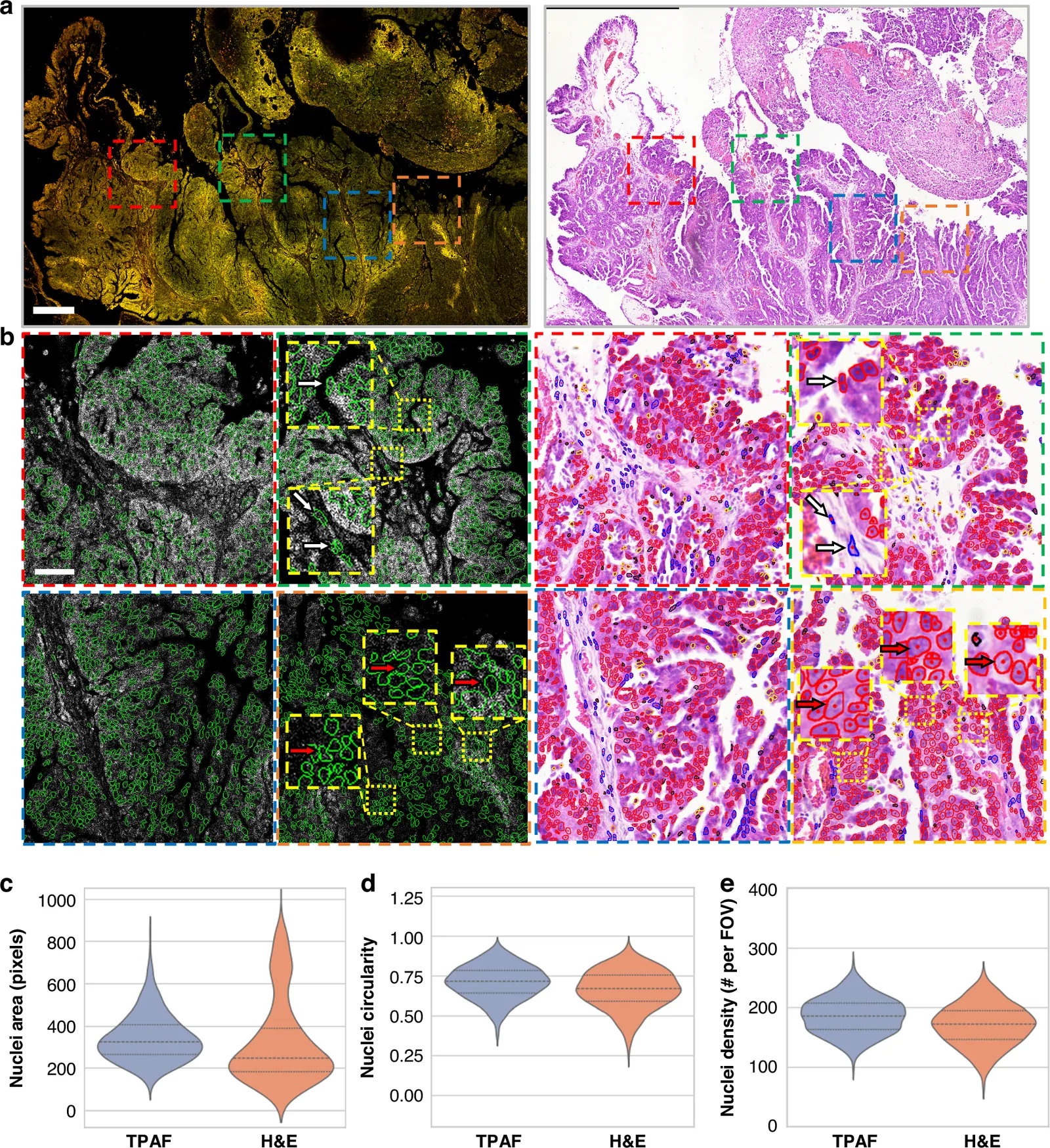
**Comparison study of nuclei segmentation in complex clinical tissue structures between AFN-DeSeg and H&E histopathology reference**. **a** Whole-slide TPAF images of ovarian cancer tissue (left) and the corresponding H&E images (right), matched regions of papillary, glandular, and stromal architectures are highlighted. Scale bar: 200 µm. **b** Zoomed-in comparisons between AFN-DeSeg segmentation on TPAF images (left) and hematoxylin-stained nuclei on H&E (right). Zoomed-in insets highlight representative morphological features: white arrows mark elongated/spindle-like stromal nuclei in the green-box ROI; red arrows mark vesicular chromatin patterns at the tumor-stroma interface in the orange-box ROI. Colored circles in the H&E panels denote HoVer-Net nuclei classifications (black: no class; red: neoplastic; green: inflammatory; blue: connective; yellow: dead; orange: non-neoplastic epithelial). Scale bar: 50 µm. Quantitative comparison of nuclear morphology metrics of (**c**) nuclear size (*N* = 200), **d** nuclei circularity (*N* = 200), and **e** nuclei count per field (randomly selected 318 × 318 µm^2^ regions, *N* = 200) between AFN-DeSeg (TPAF) and H&E across matched fields. Violin plots show strong cross-modality agreement on cancer nuclear features. All TPAF images in this figure were acquired in NADH channel (495–540 nm)

Ovarian cancer tissue exhibits highly heterogeneous microarchitecture, which poses substantial challenges for label-free segmentation due to varying cytoplasmic signal intensity. The performance of AFN-DeSeg on resected ovarian cancer tissue samples was characterized and compared with the H&E histopathology (Fig. 5). Four representative regions of interest (ROIs) in whole-slide TPAF and its matched H&E image, including papillary fronds, glandular folds, and desmoplastic stroma, highlighted by red, green, blue, and orange boxes, were selected for the comparative study (Fig. 5a).

The nuclear spatial distribution and morphology predicted by the proposed AFN-DeSeg were compared with the H&E nuclei masks (Fig. 5b). The AFN-DeSeg demonstrates its remarkable robustness to architectural variability. In papillary regions (red and green boxes), where epithelial nuclei are densely stratified and pleomorphic, AFN-DeSeg produces contours that closely track the nuclear boundaries observed in H&E image. In glandular zones (blue box), where luminal interfaces introduce abrupt signal transitions, AFN-DeSeg maintains accurate delineation without leaking into bright cytoplasmic patches. Furthermore, in stromal regions (orange box) featured with sparse and elongated nuclei embedded in collagen-rich matrices, AFN-DeSeg preserves the spindle-like shapes and avoids hallucinating boundaries in the extracellular matrix. This cross-modality agreement suggests that AFN-DeSeg relies on genuine structural cues rather than simple intensity heuristics or circularity priors. Further comparisons of TPAF and H&E images and nuclei masks were performed in representative ovarian cancer regions (papillary structure, solid tumor nests, cribriform architecture, and tumor-stroma interface, Figs. S2–S5).

The biological relevance of the segmentation was further quantitatively assessed by analyzing three key nuclear morphometric parameters i.e., nuclear size, circularity, and count per field. These metrics, critical for pathological grading, are computed using 200 FOVs (318 × 318 µm^2^) from matched TPAF and H&E regions (Fig. 5c–e). The TPAF and matched H&E regions were aligned manually using anatomical landmarks, and the matched FOVs represent approximately rather than pixel-perfectly registered regions, and the morphometric comparison is therefore performed at the level of population distributions rather than pixel-paired nuclei (Fig. 5c–e). The distribution of nuclear size obtained from TPAF images matches well with that of H&E images, confirming the morphometric accuracy of AFN-DeSeg (Fig. 5c). While the medians are comparable, nuclei in TPAF images are slightly larger on average, whereas H&E images exhibit a broader distribution with more extreme values. This discrepancy likely arises from staining processing, e.g., chemical fixation can cause nuclear aggregation to form giant nuclei or fragmentation of DNA from damaged nuclei to be small nuclear debris in H&E images. In contrast, TPAF imaging captures the in situ morphology without such artifacts. With regard to circularity (Fig. 5d), both modalities show strong alignment. The H&E images display slightly lower circularity (indicating more irregularity and atypia), which is consistent with the mechanical compression and dehydration during staining to exaggerate nuclear deformation and atypia, whereas the volumetric integrity is preserved in TPAF imaging. Furthermore, the nuclear density is highly consistent (Fig. 5e). TPAF imaging yields slightly higher counts, it suggests that AFN-DeSeg effectively resolves individual nuclei in dense clusters that might appear merged or be lost due to tissue sectioning in H&E images. These results underscore that AFN-DeSeg reliably extracts meaningful pathological nuclei features, serving as a robust option to offer a high morphological fidelity.

### Clinical quantitative diagnostics

**Fig. 6:**
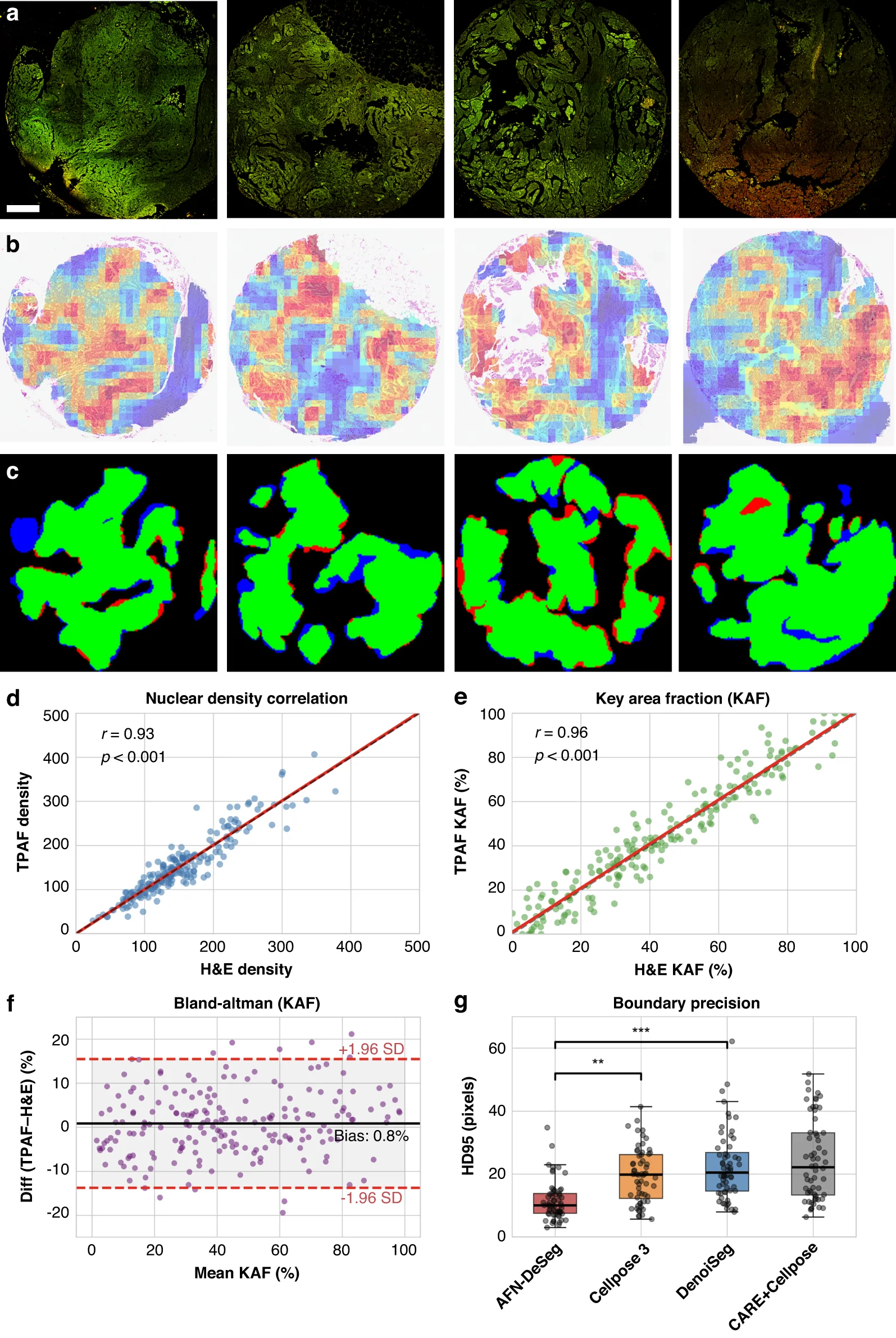
**Clinical studies of AFN-DeSeg for virtual pathology and quantitative diagnostics on ovarian cancer**. **a** Representative TMA TPAF images of tissue cores (scale bar: 600 µm). **b** Pathologist-corrected and CLAM-generated attention heatmaps, highlighting real tumor burden areas. **c** Visualization of diagnostic concordance for key area prediction. Green indicates overlap between AFN-DeSeg TPAF and H&E prediction; blue and red indicate modality-specific mismatches (TPAF: blue; H&E: red). The correlation of (**d**) Nuclear Density (*r* = 0.93) and **e** Key Area Fraction (*r* = 0.96) between TPAF prediction and H&E ground truth. **f** Bland-Altman plot of KAF. **g** Comparison of surgical margin precision using 95% HD95 (**p < 0.01, ***p < 0.001). All TPAF images in this figure were acquired in NADH channel (495–540 nm)

A study of AFN-DeSeg’s potential for clinical translation was performed using large-scale TMAs. The representative TMA TPAF images from ovarian carcinoma samples exhibit complex morphological profiles (Fig. 6a). To validate the reliability of nuclei-based diagnosis, the Key Diagnostic Area (KDA, indicating high-tumor-burden regions) with nuclear masks was predicted and compared against pathologist-corrected CLAM-generated attention heatmaps (Fig. 6b). Red regions indicate high attention scores corresponding to discriminative features of highly predictive cancer, whereas blue regions denote low-attention areas of minimal diagnostic value. And then, the KDAs are predicted from TPAF nuclei masks and H&E nuclei masks by an attention U-Net prediction model, respectively. The pixel-level concordance between KDA predictions from TPAF and H&E nuclei masks was analyzed (Fig. 6c). The blue and red areas respectively represent TPAF and H&E predictions, while green areas denote consensus. The dominated green areas indicate that the nuclei masks generated by AFN-DeSeg localizes KDA with a high sensitivity, bridging the modality gap between TPAF and H&E images. Moreover, the green areas align closely with the attention heatmap (Fig. 6b), confirming that AFN-DeSeg’s predictions are highly consistent with expert pathological assessment.

To corroborate visual observation, statistical correlation analysis (FOVs of 318 × 318 µm^2^, *N* = 200 sampled from the test set defined in Supplementary Table S5) was further performed to assess the quantitative equivalence between TPAF-derived predictions and standard histopathology (Fig. 6d–g). Nuclear density directly measures tumor cellularity. It is observed a strong linear relationship (Pearson’s *r* = 0.93, *p* < 0.001) between the TPAF counts and H&E counts for each FOV (Fig. 6d). Variance increases slightly in dense tumor regions due to nuclei aggregation, but the model remains robust without saturation. For Key Area Fraction (KAF), AFN-DeSeg achieves a high linearity of *r* = 0.96 (Fig. 6e), confirming its accuracy of quantifying tumor burden ratios. To detect potential systematic errors, the Bland-Altman analysis is performed and reveals a negligible mean bias of 0.8% with Limits of Agreement (LoA) spanning ±15% (Fig. 6f). This suggests that AFN-DeSeg does not introduce any significant systematic deviation, making it a reliable assistive tool for optical biopsy.

Finally, the precision of tumor boundary delineation was evaluated with HD95 and compared across methods (Fig. 6g). AFN-DeSeg achieves a median boundary error of ~10 pixels, significantly lower than the generalist Cellpose 3 (~20 pixels) and the joint DenoiSeg model (~21 pixels). The achieved distribution shows a long tail of outliers, which is the typical feature of challenging cases, but its significantly lower median and tighter interquartile range confirm that AFN-DeSeg provides the highest boundary precision. It is observed that these outliers arise from a small set of identifiable extreme conditions including strong autofluorescence occlusion, boundary signal dropout, mechanical tissue deformation, and TPAF-to-H&E processing discordance (Fig. S6). These residual errors are predictable and visually screenable while the median boundary precision remains representative of typical-case performance.

## Discussion

The clinical adoption of TPAF microscopy has long been hindered by the challenge of nuclear visibility with negative-contrast and the fundamental trade-off between imaging speed and quality. The future intraoperative deployment of TPAF will necessitate high-speed acquisition, which inevitably lowers photon density and degrades the visibility of critical non-fluorescent structures, specifically cell nuclei. In this work, we proposed and demonstrated the AFN-DeSeg framework to resolve this trade-off, successfully reconstructing diagnostic-grade nuclear morphology from photon-starved, noisy acquisitions of FFPE-sectioned ovarian cancer tissue. Our findings suggest that one major barrier to real-time optical biopsy is computational, and the gap between label-free optical signals and H&E-based pathology can be efficiently bridged by integrating foundation models with domain-specific restoration tasks.

The superior performance of AFN-DeSeg as compared to sequential denoising-segmentation pipelines can be attributed to two specific architectural designs: (I) the integration of DINOv3 vision transformer endows the model with a global semantic context that conventional CNNs lack. The U-Net encoders perform well at local edge detection but struggle to distinguish signal-dependent speckle noise from true texture in low-contrast regions. With the pre-trained visual priors, the transformer backbone infers the correct structural continuity of tissue architecture based on context, even when local pixel information is corrupted. It enables the model to differentiate nuclei from stochastic noise clusters, which is often missed by loss functions based solely on pixelwise loss; (II) the joint optimization strategy resolves the nuclei visibility problem by creating a task-driven feedback loop. In independent denoising tasks, the objective is pixel-wise fidelity, and the over-smoothing of nuclear boundaries often occurs since they statistically resemble background fluctuations. By incorporating segmentation loss in the optimization of the denoiser, AFN-DeSeg forces the restoration branch to preserve the edge information. To disentangle these two designs from the generic benefit of pre-training, AFN-DeSeg was benchmarked against ResNet50 baseline (Table S3). Pre-training alone already surpassed the from-scratch baselines (ΔDice ≈ 0.05 at *n* = 1 vs. Cellpose 3), yet AFN-DeSeg still outperformed ResNet50 by ΔDice ≈ 0.06 (*n* = 1) and ΔmAP ≈ 0.15 in instance detection. The residual gap is attributed to the microscopy-specific LoRA adaptation and the DINOv3 self-attention stream, which integrates global structural context across crowded fields. These features are not available for an ImageNet-pre-trained convolutional encoder alone.

Previous studies have primarily positioned TPAF microscopy either as a qualitative visualization tool or as a metabolic readout via the optical redox ratio (ORR). Here we exploit a different aspect of TPAF contrast (the negative-contrast structural signature of nuclei) and validate its utility as a quantitative morphological diagnostic dimension with the aid of AFN-DeSeg. AFN-DeSeg accurately segments nuclei across heterogeneous ovarian tissues, ranging from dense papillary clusters to sparse fibrotic stroma, demonstrating that NADH autofluorescence in TPAF images contains sufficient morphological information for label-free histopathological assessment.

It is worth noting that the proposed AFN-DeSeg is performed on FFPE-sectioned tissue rather than unfixed fresh tissue. Investigation on FFPE-sectioned tissue, which is the standard first step in computational-pathology development, enables a direct, spatially-correlated comparison with H&E-based pathology. Unfixed fresh tissue is different from the FFPE-sectioned tissue: (I) the autofluorescence intensity and corresponding spectral profile would shift in the absence of fixation-induced crosslinking; (II) tissue thickness and scattering properties are substantially different; (III) nuclear morphology in volumetric fresh tissue is 3D rather than 2D in sectioned tissue. In order to further perform intraoperative diagnosis of unfixed fresh tissue, optimized TPAF imaging system, re-calibration of the noise model on fresh-tissue, an extension of the architecture to pseudo-3D or fully volumetric processing, etc., should be further explored. Meanwhile, the denoising and resolution-enhancement function should be further explored and integrated into the proposed AFN-DeSeg to improve the diagnosis depth due to the low two-photon absorption cross-section of endogenous fluorophores and subsequently limited imaging depth^38,39^. The proposed AFN-DeSeg establishes the computational foundation for the translational study. Moreover, our system could simultaneously acquire TPAF intensity of NADH and FAD to obtain metabolic images via ORR. It is meaningful to simultaneously obtain structural and metabolic information by in vivo dual-modal diagnosis of morphological pathology and ORR using the proposed AFN-DeSeg and imaging system.

Nevertheless, the current framework is still subject to several limitations: (I) the noise model assumes a specific Poisson-Gaussian distribution with parameters calibrated to our specific PMT detectors. The primary mechanism by which generalization could fail is therefore parameter mismatch: deploying AFN-DeSeg on a different imaging system without recalibration would introduce a systematic discrepancy between the synthetic training noise distribution and the target system’s noise distribution. To evaluate how much AFN-DeSeg relies on the exact noise calibration, the noise parameters (g, σ_read_) were deliberately perturbed by ±10%, ±25%, and ±50% (Supplementary Table S1). At perturbation level up to ±25%, Dice dropped by only about 0.04 and AFN-DeSeg still outperforms every baseline, and at a large perturbation of ±50%, Dice dropped by up to ~0.12. It can be deduced that the exact calibration is not necessary, and a quick recalibration is enough for a new microscope (estimating 5–10 multi-frame FOV acquisition; Note S3); (II) TPAF imaging is inherently a 3D imaging modality, our current framework operates on 2D slices since volumetric segmentation remains computationally expensive. It is feasible to leverage inter-slice continuity to improve accuracy of 3D diagnosis, particularly for tangentially sliced nuclei, by extending AFN-DeSeg to pseudo-3D or fully volumetric architectures; (III) This study focused on ovarian cancer, but the working principle of negative-contrast segmentation is applicable to other pathologies visualized via TPAF imaging, such as gliomas and basal cell carcinomas. Future studies should evaluate the transferability of the DINOv3-based encoder to these tissue types to determine if the visual priors learned here represent universal histological features.

In summary, AFN-DeSeg provides a computational foundation for label-free morphological pathology on TPAF images, addresses a long-standing obstacle to quantitative use of two-photon autofluorescence i.e., the negative-contrast nuclear-visibility problem, and also identifies the concrete steps required to extend the framework ultimately toward intraoperative deployment.

## Materials and methods

### Datasets construction

To address the scarcity of paired TPAF data for training AFN-DeSeg, we constructed a composite dataset leveraging both public fluorescence microscopy images and in-house ovarian cancer TPAF images. The sample preparation process and imaging setup are described in Supplementary Note S1 and S2, and Fig. S7 in Supplementary Information.

#### Instrument-calibrated denoising dataset

Deep learning efficacy in microscopy is often bottlenecked by the scarcity of paired “noisy–clean” reference data. To address this issue, we curated a diverse corpus of high-quality fluorescence microscopy images to serve as noise-free references instead of relying on generic noise augmentations that fail to capture instrument-specific characteristics. We then constructed a mixed Poisson-Gaussian noise model with parameters empirically calibrated from real multi-frame TPAF acquisitions to bridge the domain gap between synthetic and real data. This instrument-calibrated physics-based model accounts for both the photon shot noise inherent to low-photon-count imaging and the electronic readout noise of the detector (see Supplementary Note S3 and Supplementary Table S4 for mathematical derivation and parameter estimation). This strategy ensures the network is trained on realistic detector-noise statistics of our two-photon imaging system while leveraging the morphological diversity of established biological datasets. The TPAF images with different noise levels are shown in Fig. S8a. AFN-DeSeg is trained jointly for denoising and segmentation on a single set of triplets, each comprises a clean reference image, a synthetically noised image, and a nuclear mask. These triplets come from two sources: public fluorescence microscopy patches and in-house ovarian cancer TPAF from cohort (II). The public images share morphological similarity with ovarian cancer TPAF images and contribute to both the denoising and segmentation objectives, though they are not ovarian cancer data; all expert annotated supervision and all quantitative evaluation come from the in-house ovarian cancer cohort. The 51 in-house FOVs of the calibration cohort (Note S2) are used solely to fit the noise model, not for training or evaluation. AFN-DeSeg is evaluated (Figs. 3–5) on a patient-disjoint held-out subset of cohort (II); the clinical key-area prediction (Fig. 6) uses the cohort (I) together with the cohort (II). Full dataset accounting is in Supplementary Table S5.

#### Human-in-the-loop segmentation label annotation

Accurate segmentation of TPAF nuclei is fundamentally complicated by the negative contrast phenomenon, where nuclear signal voids are morphologically indistinguishable from background signal dropouts. Obtaining accurate masks on such low-SNR images is challenging. To accelerate the annotation of in-house clinical TPAF data, we devised a domain-transfer strategy. We first generated high-quality reference masks from public datasets by running the standard cyto2 model on their nuclear channels. Crucially, we then paired these masks with their corresponding cytoplasm channels. Since cytoplasm fluorescence staining typically exhibits signal voids in nuclear regions, morphologically mimicking the appearance of TPAF nuclei, this created a proxy dataset for negative contrast nuclei segmentation. We used this dataset to fine-tune the cyto2 model. While this intermediate model was not fully optimized for TPAF image inference, it learned to recognize nuclear voids sufficiently to serve as an assistive tool. Consequently, for in-house TPAF data, initial masks were predicted by the fine-tuned cyto2 model and subsequently underwent rigorous manual correction with Any Labeling tool and verification by expert pathologists to ensure clinical accuracy (Fig. S8b). The final dataset was partitioned into training, validation, and testing sets following patient-level split specified in Table S5. Manual correction was performed only on the clean 16-frame-averaged reference images instead of the noisy images at different levels. Because the underlying nuclei were the same biological objects across different noise levels, only the noise contamination of the input TPAF image differed, enabling the corrected masks serve as the ground truth for all noise levels. During training, synthetic noise was instantaneously added to the clean reference images, while the segmentation ground truth remained the same human-corrected mask. The average annotation time was approximately 20 min per 512 × 512 FOV, corresponding to a total human annotation effort of approximately 100 h across all ~300 FOVs (~130,000 nuclei instances).

To quantify the magnitude of human input in this pipeline, the agreement between the raw fine-tuned cyto2 predictions and the final human-corrected masks was retrospectively analyzed. The mean Dice coefficient between them was 0.72 across all annotated FOVs, indicating that corrections were substantial rather than cosmetic. The corrections predominantly comprised three types of 38.5% adding missed nuclei in low-contrast regions, 16.2% removing false positives in bright cytoplasmic patches and vesicles, and 32.6% reshaping boundaries of irregularly shaped tumor nuclei. Annotations were performed by a single trained annotator. All corrected masks were subsequently reviewed by a board-certified pathologist, with 13.4% of FOVs flagged for a second round of correction. Per-pipeline statistics are summarized in Supplementary Table S2.

#### Clinical test set and histopathology benchmarks

Patient cohort and inclusion/exclusion criteria: This study is based on two independent patient cohorts: (I) a TMA cohort (130 TMA images from 68 HGSOC patients), (II) a WSI cohort (6 WSI images from 6 patients). The two cohorts are mutually exclusive at the patient level, and (III) a separate 4-patient calibration cohort, used solely for noise-model fitting and not for any experiment described here, is described in Note S1 and Supplementary Table S5. Full cohort definitions, sample sizes, inclusion/exclusion criteria, and acquisition protocols are reported in Supplementary Note S1; a consolidated dataset summary is in Supplementary Table S5. All patients were recruited under ethics approval No. KS22335 (Shanghai First Maternity and Infant Hospital). The in-house ovarian cancer H&E TMA and WSI images are first manually cropped to exclude as much empty background as possible. Then, Hover-Net nuclei segmentation and classification model pre-trained on PanNuke dataset was applied to generate H&E nuclei segmentation masks as a computational reference standard of nuclear morphology and statistics for evaluating the clinical accuracy and applicability of TPAF nuclei segmentation masks^40^. We also trained an ovarian cancer diagnosis model based on an attention-based multiple instance learning (ABMIL) model named CLAM with UBC-OCEAN dataset consisting of 500 weakly labeled ovarian cancer TMA and WSI images^41^. The CLAM model generated a heatmap of each in-house ovarian cancer H&E image, indicating the key pathological areas for diagnosis. The training and inference procedures are described in Supplementary Note S4.

### Implementation and training of AFN-DeSeg

#### Model architecture

##### Input channel

The TPAF of NADH was recorded as the input for AFN-DeSeg, since the NADH channel has stronger intensity and better SNR than the FAD channel under excitation at 860 nm. The FAD channel carries complementary information but was not used; AFN-DeSeg requires only the single NADH channel to perform morphological pathology.

To reconcile the conflicting demands of denoising and segmentation, the AFN-DeSeg model employs a dual-stream encoder architecture designed to process a single noisy TPAF image patch (512 × 512 pixels) and simultaneously output a denoised intensity image and a nuclear segmentation mask.

##### Dual-encoder backbone

The framework consists of two parallel feature extraction branches. A U-Net-based encoder comprises four down-sampling blocks. Each block contains two 3 × 3 convolutional layers (padding=1) with Batch Normalization (BN) and ReLU activation, followed by 2 × 2 max pooling. This branch extracts high-frequency morphological features, yielding a bottleneck feature map of 32 × 32 × 512. The other branch is a Vision Transformer (ViT-Base) initialized with pre-trained DINOv3 weights. It utilizes a patch embedding layer with patch size of 16 × 16 resulting in a 32 × 32 token grid with a hidden dimension of 768. To adapt the ViT to the specific TPAF domain, we apply LoRA to 12 layers of Multi-Head Self-Attention (MHSA), which allows efficient finetuning of the semantic features.

##### Feature fusion and decoders

To align the heterogeneous feature maps, the ViT output patch tokens are reshaped to a dimension of 32 × 32. These features (768 channels) are then concatenated with the U-Net bottleneck features (512 channels) along the channel dimension. The combined feature map (32 × 32 × 1280) is then fused and compressed via a Fusion Bottleneck Block, which successively consists of a 1 × 1 convolution (output channels = 512), Batch Normalization, ReLU, 3 × 3 convolution (output channels = 512, padding = 1), Batch Normalization, ReLU. No dropout is applied within the block. This unified representation feeds into two task-specific decoders.

##### Joint optimization of denoising and segmentation branches

Both decoders are initialized using Kaiming initialization and share a symmetric structure. The denoising decoder consists of four up-sampling blocks using bilinear up-sampling followed by convolution. Skip connections from the U-Net encoder are added to corresponding decoder layers. It outputs a single-channel continuous intensity map. Similarly, the segmentation decoder utilizes skip connections to restore boundary precision before terminating with a 1×1 convolution and sigmoid activation to generate a pixel-wise probability map. During training, the Fusion module and shared encoders are updated via backpropagation of the total composite loss, ensuring that the shared representation captures both structural fidelity and semantic discriminability. The complete architecture of AFN-DeSeg is shown in Fig. S9.

#### Loss design

The weighting factors are empirically tuned to balance the gradient magnitudes of the heterogeneous tasks. Since the pixel-wise reconstruction loss ${L}_{{rec}}$ on normalized images tends to be numerically small (~10^−2^), we assign a higher weight to the segmentation loss (${\lambda }_{{seg}}=5.0$) to prevent the restoration objective from dominating the optimization objective. Conversely, the perceptual loss is weighted lower (${\lambda }_{{\mathrm{percep}}}=0.1$) to act as a soft constraint to suppress high-frequency artifacts. The final configuration is set to ${\lambda }_{{\mathrm{rec}}}=1.0$, ${\lambda }_{{\mathrm{seg}}}=5.0$ and ${\lambda}_{{\rm{percep}}}=0.1$, ${L}_{{\mathrm{total}}}={\lambda }_{{\mathrm{rec}}}{L}_{{\mathrm{rec}}}+{\lambda }_{{\mathrm{seg}}}{L}_{{\mathrm{seg}}}+{\lambda }_{{\mathrm{percep}}}{L}_{{\mathrm{percep}}}$. The reconstruction loss (${L}_{{\mathrm{rec}}}$) is defined as the pixel-wise Mean Absolute Error (L1 loss) between the predicted denoised image and the synthetic noise-free ground truth. The segmentation loss (${L}_{{\mathrm{seg}}}$) is calculated as the sum of the Dice Coefficient (DC) loss and Binary Cross-Entropy (BCE) loss. This combination addresses the class imbalance in sparse nuclear targets. The perceptual loss (${L}_{{\mathrm{percep}}}$) is defined as the Mean Squared Error (MSE) between feature maps extracted from the predicted image and the ground truth image using a frozen cyto2 segmentation model. Features are extracted from the bottleneck and the first up-sampling layer. Note that inputs to the perceptual network are z-score normalized to match the cyto2 training distribution.

#### Training

The training pipeline consisted of two stages: microscopy domain adaptation and joint denoising and segmentation optimization.

##### Microscopy domain adaptation

To adapt the DINOv3 backbone to the TPAF domain, we first performed self-supervised training on a dataset of approximately 4500 unlabeled TPAF patches. We applied LoRA with a rank *r* = 16 and *α* = 16 to the Query and Value matrices of the MHSA blocks. The network was optimized for 50 epochs using the native DINO student-teacher distillation objective. Crucially, the optimization was driven by enforcing consistency across augmented views (multi-crops and intensity perturbations) of the same patch. The teacher network was updated via Exponential Moving Average (EMA) with a decay rate of 0.996 to stabilize the semantic target generation.

##### Joint denoising and segmentation optimization

The full AFN-DeSeg framework was trained end-to-end using a joint optimization strategy. This created a mutual reinforcement mechanism, the segmentation objective acted as a semantic constraint to prevent over-smoothing, while the restoration objective clarified the input for the segmentation head. Gradients from both the restoration and segmentation tasks were backpropagated simultaneously to update the shared dual-encoder backbone. We used the AdamW optimizer with a weight decay of 0.05 and gradient clipping (max norm = 1.0) to stabilize the training dynamics and prevent gradient explosion in the Transformer branch. The learning rate was initialized at 10^−4^ and followed a cosine annealing schedule, decaying to 10^−6^. The batch size was set to 4. The model was trained for 150 epochs. Early stopping was triggered if the validation segmentation Dice did not improve for 20 consecutive epochs, the Dice alone (not a composite of segmentation and denoising metrics) was used because it is a more sensitive indicator of joint-optimization convergence in our setting. Input images underwent geometric augmentations including random flips and orthogonal rotations. Crucially, to align the monochromatic TPAF data with the pre-trained feature space of the DINOv3 backbone, which expects RGB inputs, single-channel TPAF patches were replicated along the channel dimension. These pseudo-RGB inputs were then normalized to [0, 1] and strictly standardized using ImageNet statistics (mean=0.485, 0.456, 0.406; std=0.229, 0.224, 0.225) to preserve the manifold of the pre-trained weights. Domain-specific normalization statistics could be explored in future work as a residual design choice.

### Benchmark methods

To rigorously evaluate the performance of AFN-DeSeg, we benchmarked it against six representative methods covering distinct computational paradigms, including BM3D, CARE, Noise2Fast, DenoiSeg, Cellpose 3, and ResNet50 baseline to disentangle the contribution of pre-trained representations from the contribution of our dual-encoder architecture (see Sections “Evaluation of AFN-DeSeg denoising performance on TPAF images of ovarian cancer tissue” and “Evaluation of AFN-DeSeg nuclei segmentation performance on TPAF images of ovarian 193 cancer tissue” and Discussion). All learning-based benchmarks shared the identical training, validation, and test splits as AFN-DeSeg, and were trained on synthetic noisy-clean pairs generated using the same instrument-calibrated MPG noise model. Detailed network architectures, hyperparameter settings (learning rates, batch sizes, epochs), and implementation protocols for each method are comprehensively documented in Supplementary Note S5, which also gives the full ResNet50 specification (trainable parameters matched to AFN-DeSeg within a factor of two).

### Key diagnostic area prediction with nuclei masks

To demonstrate the clinical utility of the segmented nuclei masks, we designed a supervised model to identify histologically relevant regions solely based on the spatial distribution and morphology of nuclei. The model took binary nuclei masks, generated from either TPAF or H&E images, as input and generated a probability map of diagnostic key areas. Ground truth labels were derived from attention heatmaps generated by CLAM computational pathology model on corresponding H&E slides, subsequently refined by expert pathologists to ensure clinical relevance.

An Attention U-Net^42^ was employed to augment the standard U-Net backbone with attention gates (AGs) at skip connections. This mechanism allowed the network to selectively focus on salient sparse features within non-uniform nuclear distributions without requiring excessive model complexity (Fig. S10).

The dataset consisted of 130 TMAs and 6 WSIs defined in Section “Clinical test set and histopathology benchmarks” (74 patients), among which 30 TMAs and 2 WSIs were used for training and validation, and 100 TMA cores and 4 WSIs were used for testing. All images were partitioned into 512 × 512 patches. The network was trained using the Adam optimizer with a hybrid Dice-BCE loss function for 100 epochs.

### Evaluation metrics

To comprehensively assess the performance of AFN-DeSeg, we employed an evaluation protocol covering image restoration fidelity, segmentation precision, nuclei morphological quantification, and downstream clinical diagnostic utility.

#### Image restoration metrics

The quality of denoised TPAF images was quantified using Peak Signal-to-Noise Ratio (PSNR) and Structural Similarity Index (SSIM). These metrics measure pixel-level reconstruction error and structural preservation.

#### Segmentation metrics

To evaluate the algorithmic performance of nuclear detection, we utilized two key indicators. DC measured the global pixel-wise overlap between predicted masks and ground truth annotations. Mean Average Precision (mAP @ IoU 0.5) assessed instance-level detection performance.

#### Nuclei morphological metrics

Beyond pixel overlap, it is critical to ensure that TPAF-derived nuclei preserve statistically accurate pathological features. We quantified three key morphological descriptors across 200 paired FOVs of 318 × 318 ${\mu m}^{2}$. Nuclear Area measured the size distribution of individual nuclei. Nuclear Circularity quantified nuclear atypia and pleomorphism (a hallmark of malignancy). Nuclear Density counted the number of segmented nuclei per unit tissue area. This metric serves as a primary indicator of tumor cellularity.

#### Clinical validation metrics

To assess the diagnostic interchangeability between TPAF-based and H&E-based assessments, different metrics were employed. Pathological Feature Correlations incorporated linear regression analysis and Pearson’s correlation quantified the concordance of Nuclear Density and Key Area Fraction (KAF) between TPAF predictions and H&E reference. Bland-Altman Analysis detected systematic bias and evaluated the Limits of Agreement (LoA) for diagnostic indices. Boundary Precision (HD95) measured the 95% Hausdorff Distance to assess surgical margin precision.

Statistical independence note: The *N* = 500 (Sections “Evaluation of AFN-DeSeg denoising performance on TPAF images of ovarian cancer 155 tissue”–”Evaluation of AFN-DeSeg nuclei segmentation performance on TPAF images of ovarian 193 cancer tissue”) and *N* = 200 (Section “Comparative evaluation of AFN-DeSeg with H&E histopathology”) are the held-out test FOVs of cohort (II): the ~200 FOVs contributed by the 4 patients that are entirely excluded from AFN-DeSeg training. The *N* = 500 are 256 × 256 patches cropped from these held-out FOVs, and the *N* = 200 are the corresponding 512 × 512 FOVs (with matched H&E). AFN-DeSeg is trained and tested only on cohort (II) TPAF FOVs and not on any TMA core (cohort I); the calibration cohort (III) is used only to fit the MPG noise model and is excluded from all training, validation, and test sets. The three cohorts are mutually exclusive at the patient level (Section “Clinical test set and histopathology benchmarks”, Note S1). Detailed metric definitions are in Supplementary Note S6.

### Computational environment

All computational experiments, including data synthesis, model training, and inference, were executed on Google Colab and a computing workstation with an NVIDIA GeForce RTX 3090 GPU, an Intel Core i9 Central Processing Unit (CPU), and 64 GB of system RAM. All codes are implemented in Python, with necessary libraries.

## Supplementary information

SUPPLEMENTAL MATERIAL

## Data Availability

The raw data reported in this study are available from the corresponding author on reasonable request. The structure of the AFN-DeSeg model and key diagnostic area prediction model, the dataset construction process, and additional visualization results are in Supplementary Information file. The codes and testing data for training, inference and evaluation with AFN-DeSeg are available through Github (https://github.com/z-pan/AFN-DeSeg).
