# Supplementary material for: Label-free ovarian cancer histopathological diagnosis using two-photon autofluorescence microscopy with a joint denoising and segmentation framework
Source: Light Sci Appl. 2026 Aug 27;15:366. doi: 10.1038/s41377-026-02464-6 (PMC13518896; doi:10.1038/s41377-026-02464-6)
Supplement: Supplementary file 1 [file 41377_2026_2464_MOESM1_ESM.pdf]

Supplementary Information for

Label-free ovarian cancer histopathological diagnosis using two-photon autofluorescence microscopy with a joint denoising and segmentation framework

Zhengyuan Pan[1], Naikun Song[1], Shanshan Cheng[2], Wen Pang[2], Hongen Liao[1,*], Yu Wang[2*], Bobo Gu[1,3*]

[1]School of Biomedical Engineering, Shanghai Jiao Tong University, Shanghai 200030, China

[2]Department of Gynecology, Shanghai First Maternity and Infant Hospital, School of Medicine, Tongji University, Shanghai 200092, China.

[3]Shanghai Key Laboratory of Flexible Medical Robotics, Tongren Hospital, Institute of Medical Robotics, Shanghai Jiao Tong University, Shanghai, China

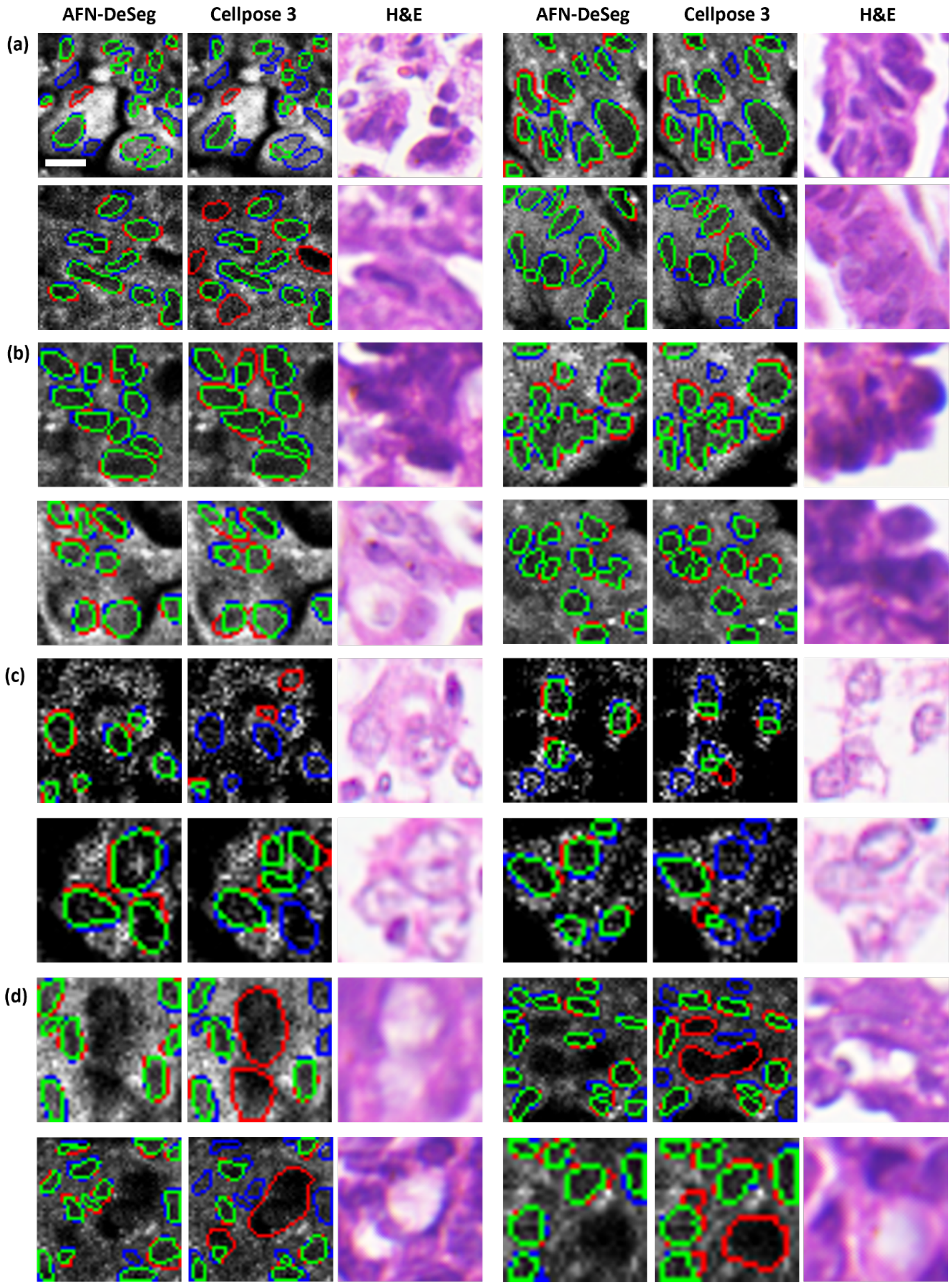

Figure S1. The segmentation performance on common nuclear features and corresponding H&E reference. Red: segmentation masks of AFN-DeSeg or Cellpose 3; Blue: GT segmentation mask; Green: correct segmentation region. GT nuclei mask (blue), prediction nuclei mask (red), and overlapping areas of prediction and GT (green) are overlaid on the denoised images for visual comparison. Scale bar: 20 µm. (a) Nuclear atypia. (b) Aggregated and adjacent nuclei. (c) Vesicular chromatin. (d) Non-nuclei vesicles. All TPAF images in this figure were acquired in NADH channel (495-540 nm).

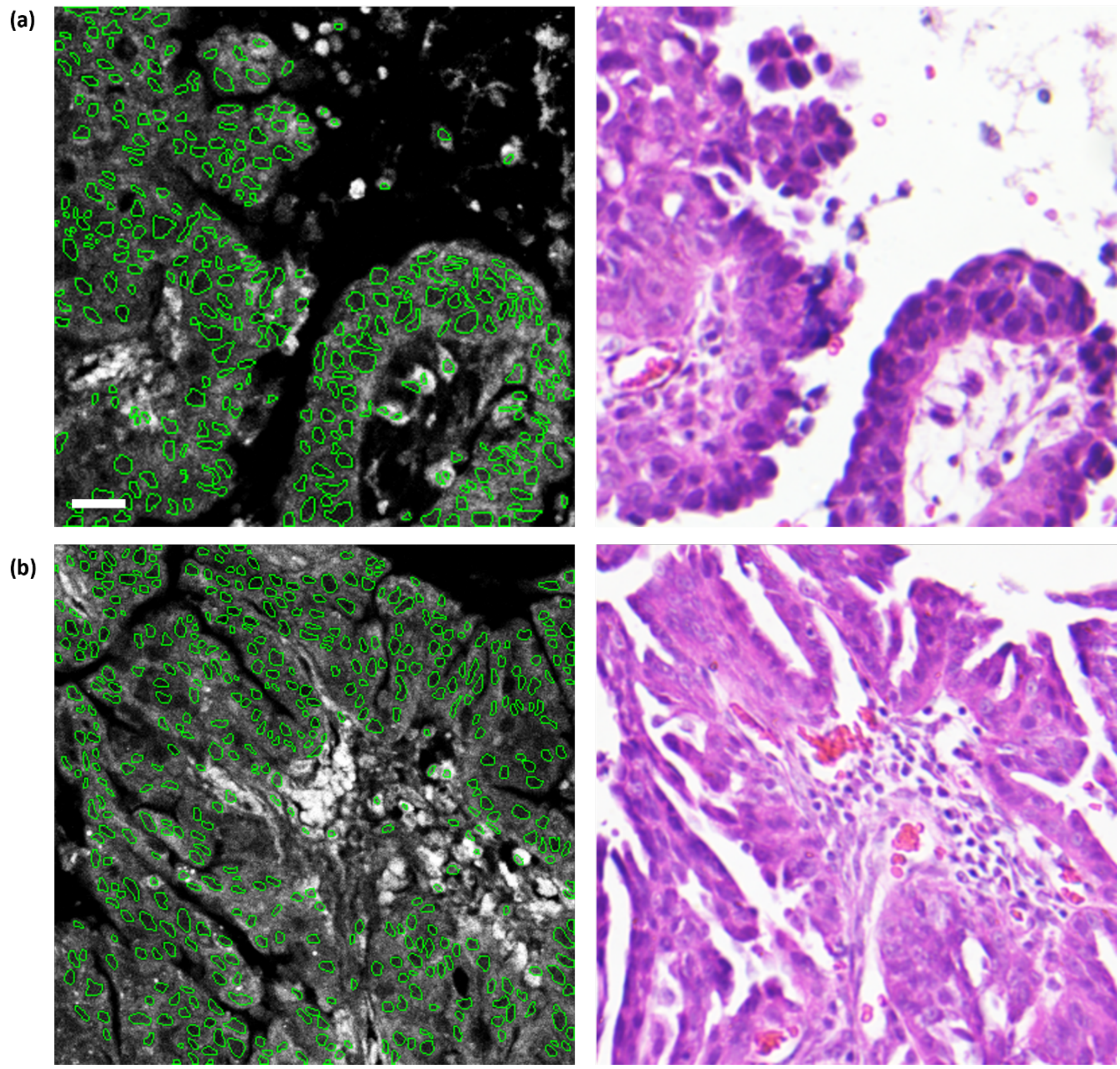

Figure S2. AFN-DeSeg nuclei segmentation masks on papillary structure of TPAF ovarian cancer images, and corresponding H&E reference. GT nuclei mask (blue), prediction nuclei mask (red), and overlapping areas of prediction and GT (green) are overlaid on the denoised images for visual comparison. Scale bar: 50 μm. (a) and (b) are from different ovarian cancer samples. All TPAF images in this figure were acquired in NADH channel (495-540 nm).

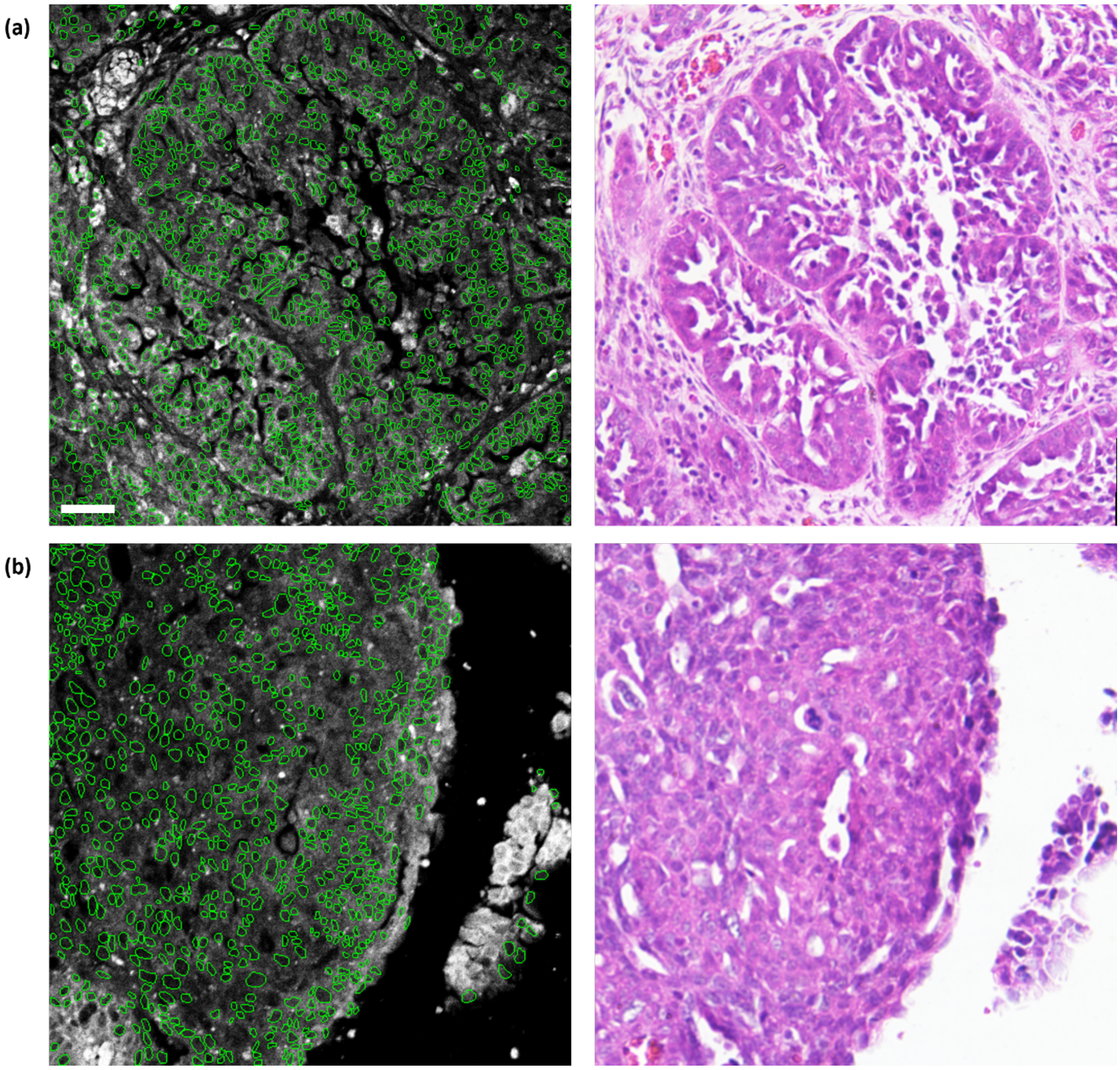

Figure S3. AFN-DeSeg nuclei segmentation masks on solid nests structure of ovarian cancer images, and corresponding H&E reference. GT nuclei mask (blue), prediction nuclei mask (red), and overlapping areas of prediction and GT (green) are overlaid on the denoised images for visual comparison. Scale bar: 150 µm. (a) and (b) are from different ovarian cancer samples. All TPAF images in this figure were acquired in NADH channel (495-540 nm).

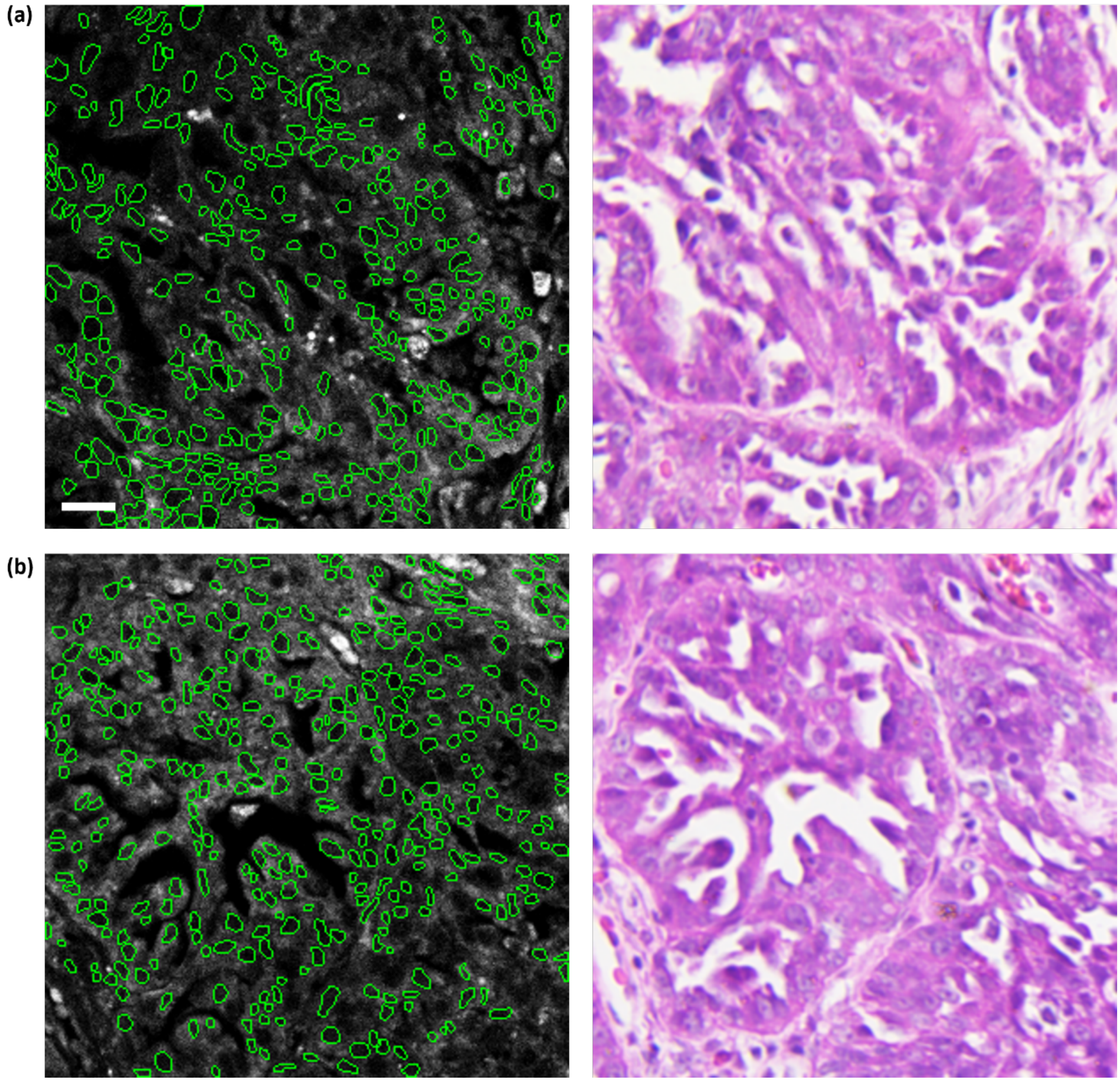

Figure S4. AFN-DeSeg nuclei segmentation masks on cribriform architecture of ovarian cancer images, and corresponding H&E reference. GT nuclei mask (blue), prediction nuclei mask (red), and overlapping areas of prediction and GT (green) are overlaid on the denoised images for visual comparison. Scale bar: 50 µm. (a) and (b) are from different ovarian cancer samples. All TPAF images in this figure were acquired in NADH channel (495-540 nm).

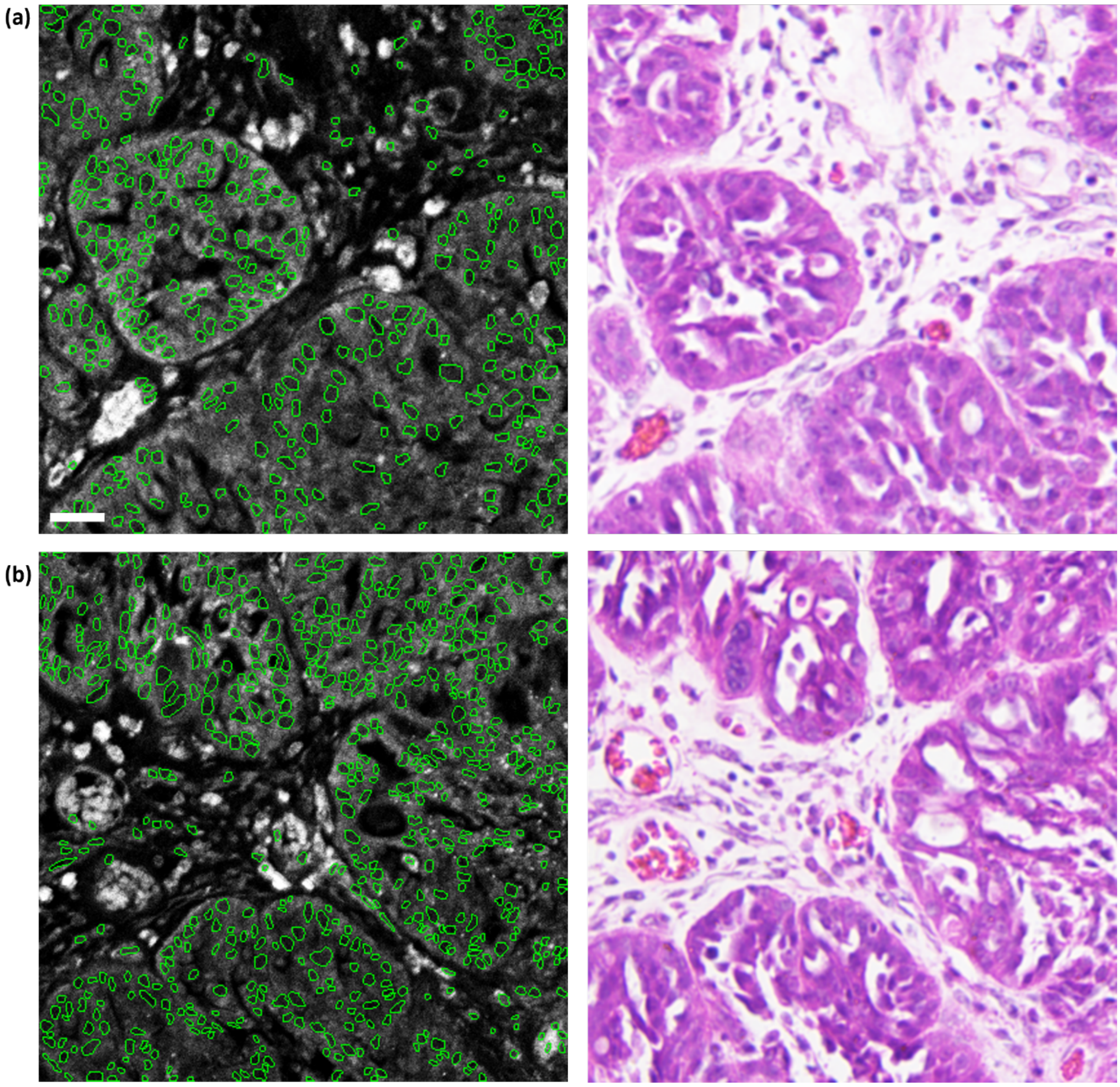

Figure S5. AFN-DeSeg nuclei segmentation masks on tumor-stroma interface of ovarian cancer images, and corresponding H&E reference. GT nuclei mask (blue), prediction nuclei mask (red), and overlapping areas of prediction and GT (green) are overlaid on the denoised images for visual comparison. Scale bar: 100 µm. (a) and (b) are from different ovarian cancer samples. All TPAF images in this figure were acquired in NADH channel (495-540 nm).

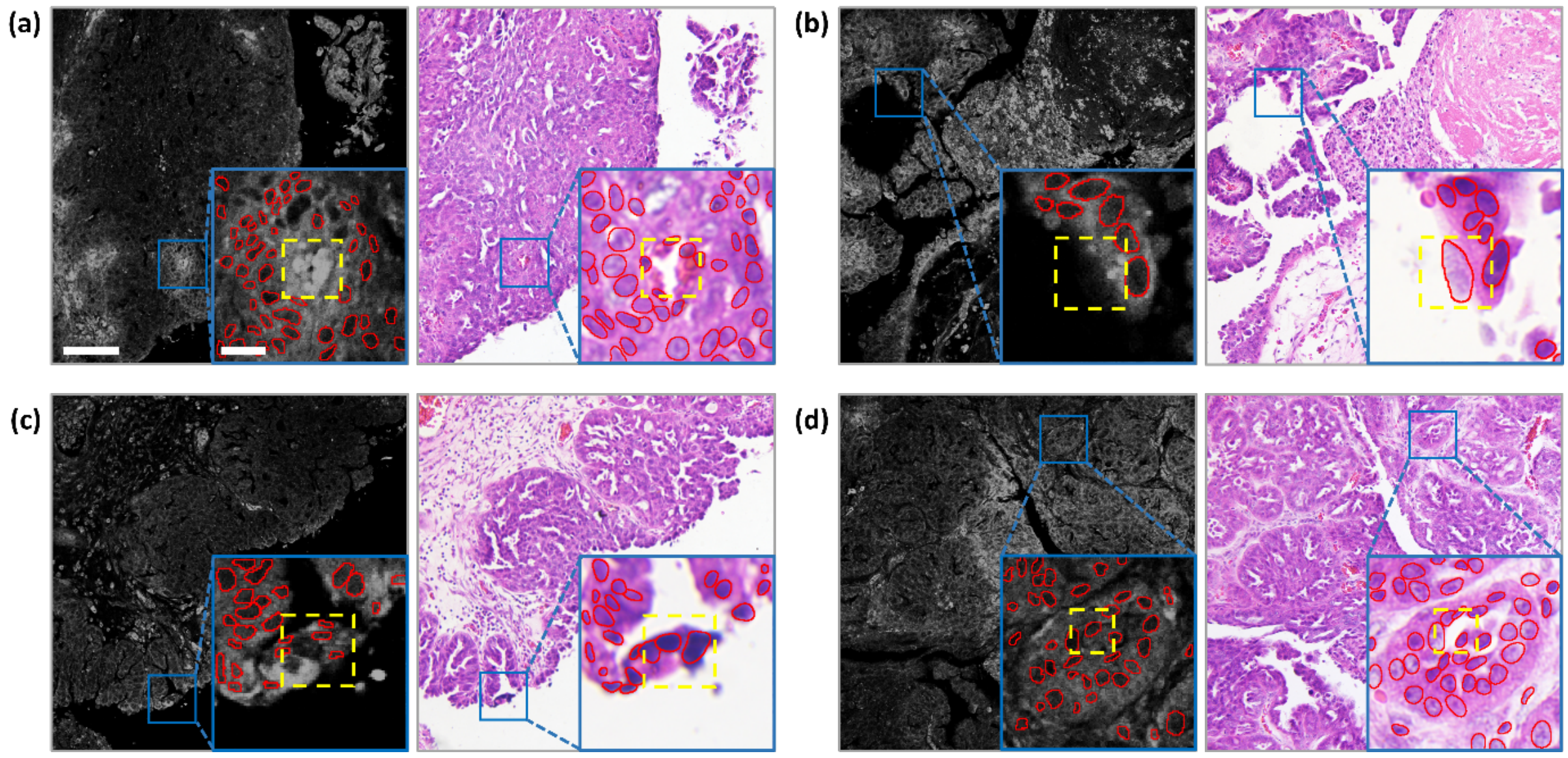

Figure S6. Representative failure cases of histopathological diagnosis using AFN-DeSeg. (a) strong autofluorescence occlusion, (b) boundary signal dropout, (c) mechanical tissue deformation, and (d) TPAF-to-H&E processing discordance (false positive). Four categories of failure modes were identified by ranking held-out test FOVs by HD95 (highest first) and Dice (lowest first); each lettered panel shows one representative case. Within each panel, the grayscale image (left) is the TPAF overlaid with AFN-DeSeg nuclear contours and the color image (right) is the spatially matched H&E reference; blue boxes mark the magnified regions (insets) and the yellow dashed box marks the failure region. Red contours denote segmented nuclei (TPAF: AFN-DeSeg prediction; H&E: reference segmentation). Scale bar: 100 µm, 20 µm (zoomed-in regions).

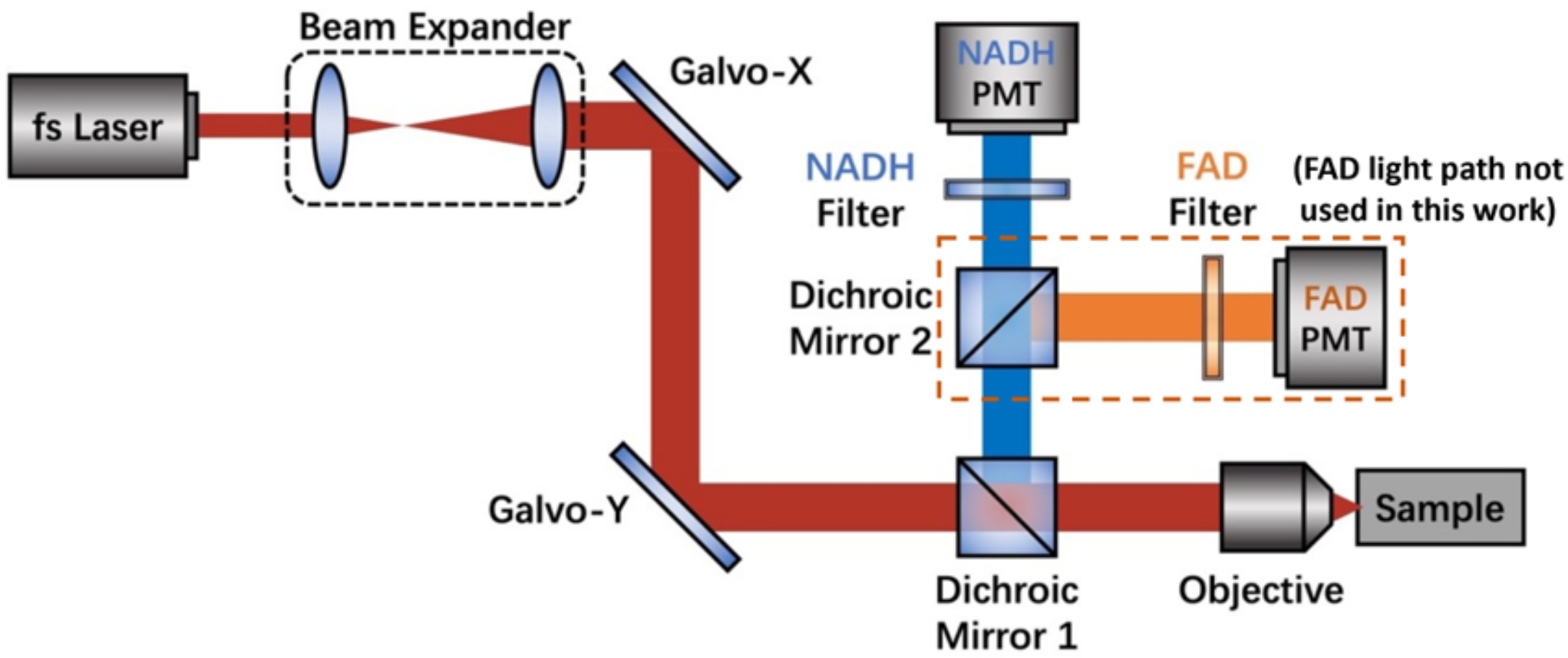

Figure S7. Diagram of TPAF imaging setup. The excitation light (860 nm) from the Ti:Sapphire femtosecond laser source transmits through X-Y galvo scanners and optical lenses to the sample. The excitation beam (red) is reflected by the first dichroic mirror (DM1) into the objective. The excited fluorescence emission is reflected by DM1 and split by DM2 into NADH (blue) and FAD (green) detection channels. Each channel is equipped with a specific emission filter (FAD: 575-645 nm; NADH: 495-540 nm). The FAD detection channel is shown for completeness, but was not used in this work; only the NADH channel (495–540 nm) was acquired as input to AFN-DeSeg. PMT: Photomultiplier tube, DM: Dichroic Mirror.

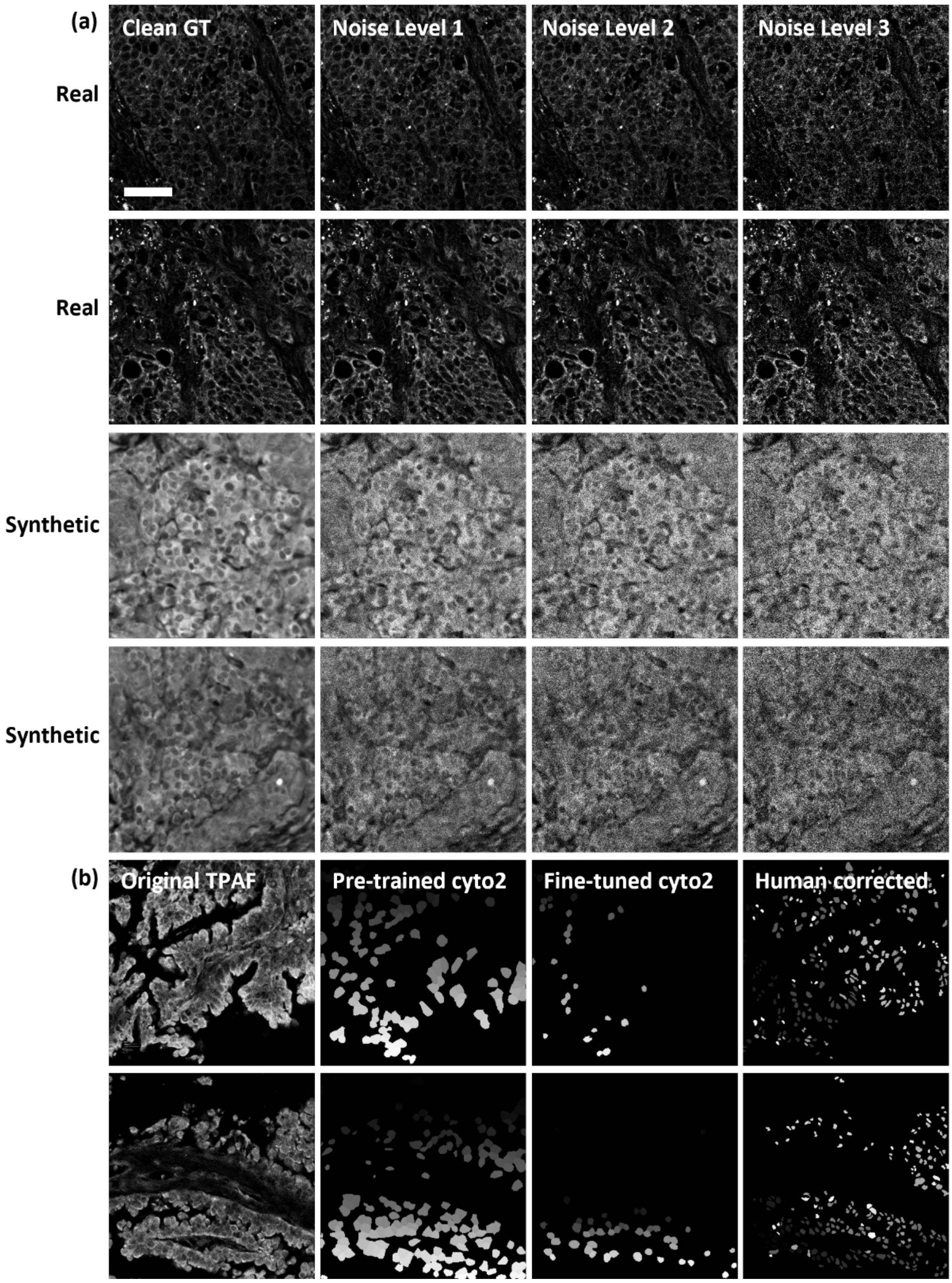

Figure S8. Sample data from denoising and segmentation train/validation/test datasets. (a) Data for denoising task. Row 1 and Row 2 show real noisy TPAF images acquired at four frame-averaging conditions (n = 1, 4, 8, 16 frames; left to right), the TPAF image in the rightmost column serves as the noise-free reference. Row 3 and Row 4 show synthetic noisy images obtained by applying the instrument-calibrated MPG noise model (Note S3) to noise-free reference images from the NeurIPS 2022 Cell Segmentation Challenge dataset. Scale bar: 50 µm. (b) The segmentation masks of the pre-trained cyto2 model, the fine-tuned cyto2 model, and the masks with human correction.

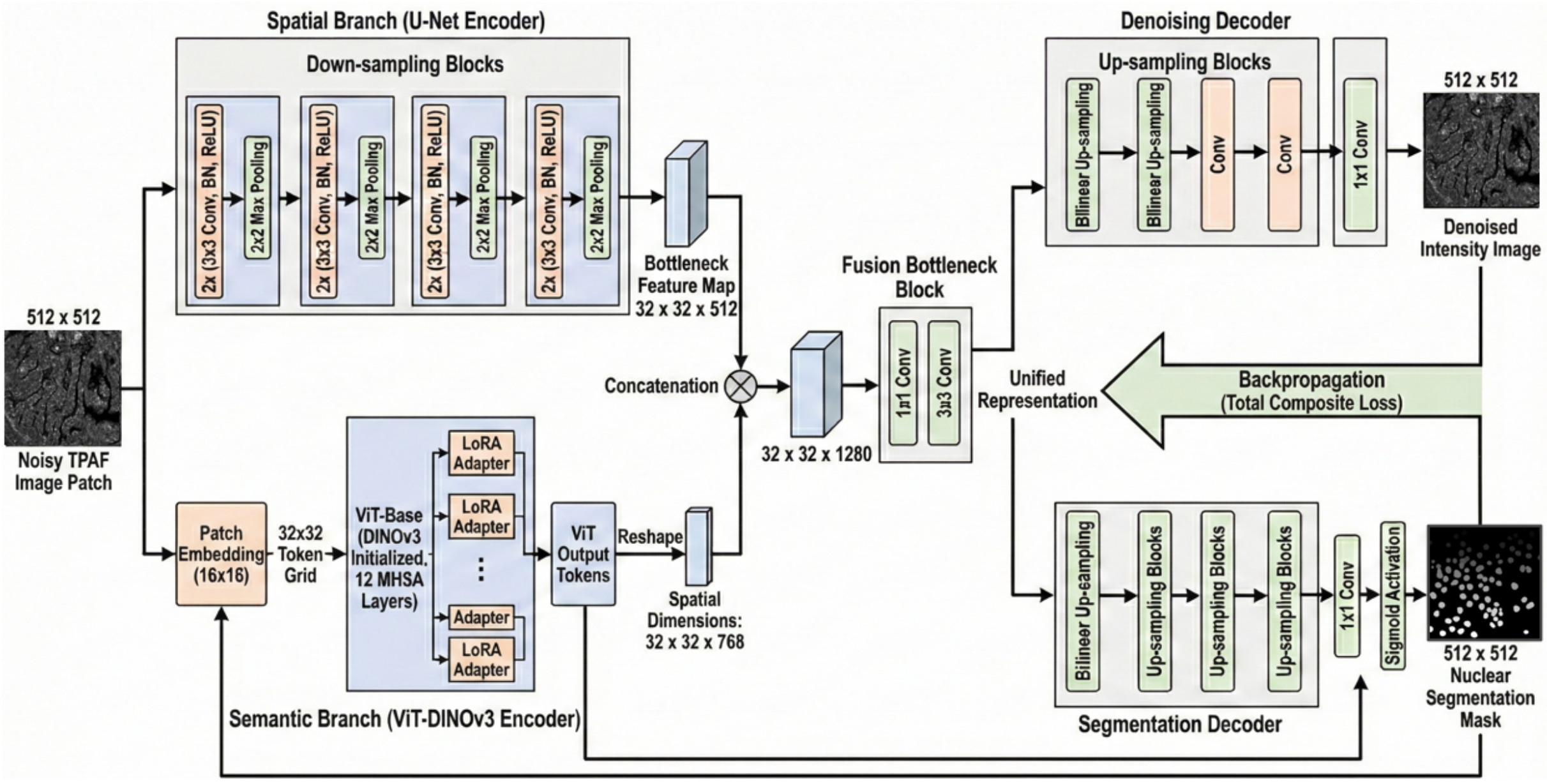

Figure S9. The framework processes a noisy TPAF input through a dual-stream encoder. The U-Net captures high-frequency morphological details via four down-sampling blocks, while DINOv3 extracts global context. Feature maps from both branches are aligned, concatenated, and fused into a unified representation via a Fusion Bottleneck Block (successively 1×1 convolution, BatchNorm, ReLU, 3×3 convolution with padding 1, BatchNorm, ReLU; output channels = 512; no dropout). This shared representation drives two parallel task-specific decoders: a denoising decoder outputting a restored intensity image and a segmentation decoder generating a nuclear mask. Crucially, both decoders employ bilinear up-sampling and utilize skip connections (not shown) from the U-Net branch to restore fine-grained boundary precision. The entire network is optimized jointly, with the total composite loss backpropagated to update the fusion module and both encoders.

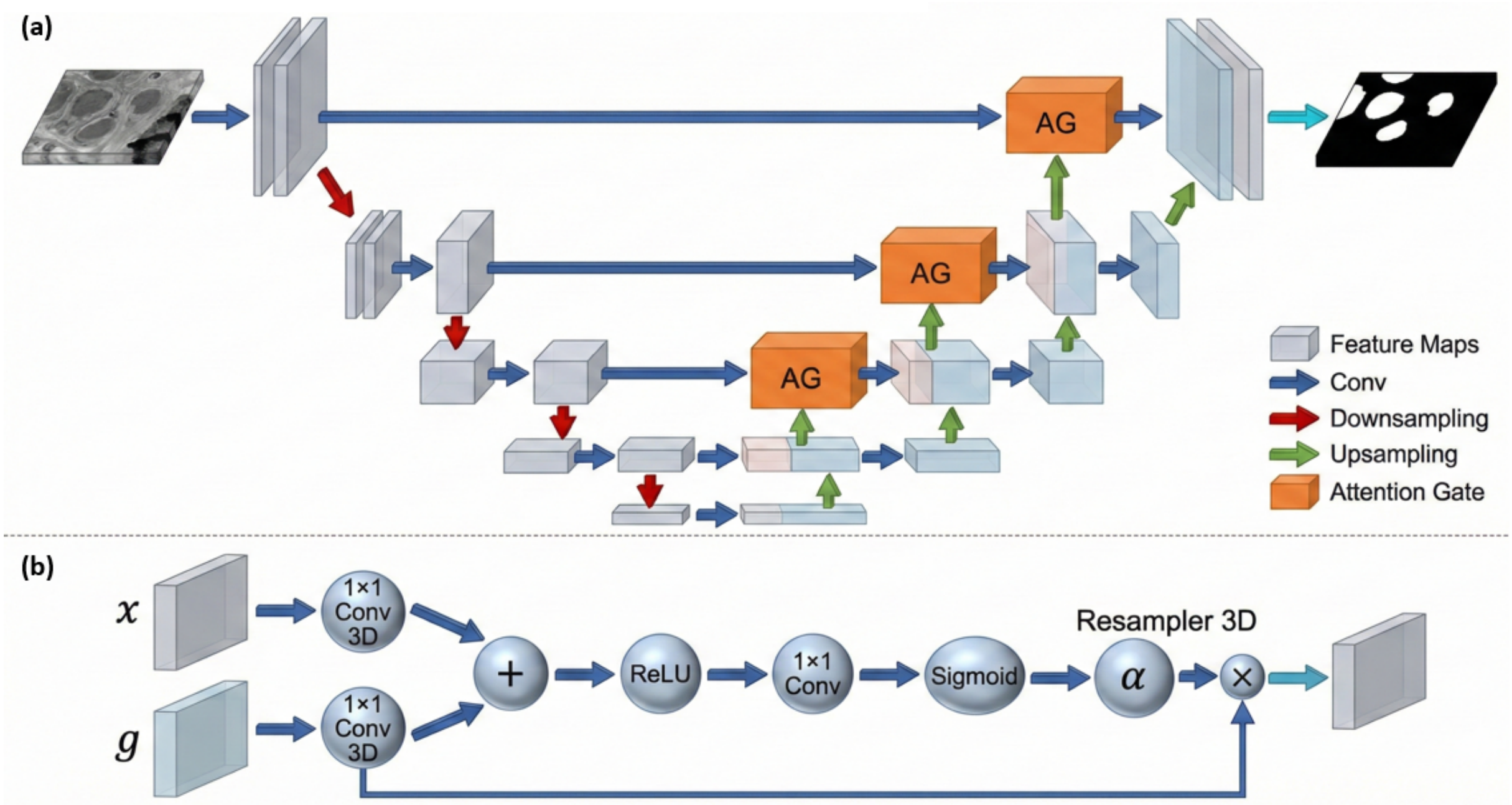

Figure S10. Design of attention U-Net for key diagnostic area prediction. (a) Schematic representation of the network architecture. The model accepts binary nuclei masks as input and utilizes a U-shaped encoder-decoder structure. The encoder (left) captures high-level morphological features through successive convolutional and down-sampling operations (red arrows). The decoder (right) reconstructs the spatial resolution via up-sampling (green arrows). Crucially, standard skip connections are replaced by Attention Gates (AGs) (orange blocks), which filter the encoder features before they are concatenated with the decoder features. (b) Detailed mechanism of the Attention Gate. The AG receives the feature map (x) from the encoder and the gating signal (g) from the decoder. These inputs undergo linear transformations (1×1 convolutions), element-wise addition, and non-linear activation (ReLU, Sigmoid) to compute attention coefficients. These coefficients are multiplied element-wise with the original input to suppress irrelevant background regions and highlight salient nuclear distributions for the final probability map output.

Note S1. Sample collection and preparation

All human tissue samples were collected from patients who underwent ovarian tumor resection, with approval by the Institutional Ethics Committee of Shanghai First Maternity and Infant Hospital with written informed consent (approval No. KS22335).

Cohort details. The tissue resource comprises three biologically independent patient cohorts.

(I) TMA cohort (Section 2.5, Fig. 6): 130 paired TPAF/H&E TMA cores from 68 HGSOC patients. Used exclusively for the clinical key-area prediction pipeline (key diagnostic area Attention U-Net training on a 30-core, 2-WSI subset; testing on the remaining 100 cores, 4 WSIs); AFN-DeSeg itself is not trained on any TMA core.

(II) WSI cohort (Sections 2.2-2.4; Fig. 3-5): Six patients (6 whole-slide images) provided ~300 human-annotated 512×512 TPAF FOVs (Supplementary Table S2). The ~100 FOVs from 2 of these patients, and the public dataset images, are used for training AFN-DeSeg (~2,500 patches; 10% randomly held out as a validation set for early stopping). The ~200 FOVs from the remaining 4 patients form a patient-disjoint held-out test set: ~800 256×256 FOVs (159×159 μm^2) are cropped from them, 500 of which are used for the denoising and segmentation metrics of Fig. 3-4, and the 200 full 512×512 FOVs (318×318 μm^2, with matched H&E) are used for the morphometric comparison of Fig. 5 (N = 200). Training and testing FOVs are therefore patient-level independent.

(III) Calibration cohort (noise model fitting only): Four additional ovarian cancer patients contributed 51 unique FOVs acquired at four frame-averaging levels (n = 1, 4, 8 as noisy inputs; n = 16 as the noise-free reference). These FOVs are used solely to calibrate the mixed Poisson–Gaussian noise-model parameters (g, σread) reported in Table S4. They do not enter the AFN-DeSeg training, validation, or test sets, nor any figure's quantitative results.

All three cohorts are mutually exclusive at the patient level (pairwise 0 overlap).

Inclusion criteria: primary ovarian tumor resection; histologically confirmed HGSOC; age ≥ 18 years; signed informed consent.

Exclusion criteria: prior neoadjuvant chemotherapy; insufficient tumor content (< 20%); significant autolysis or processing artifacts.

After excision, the resected ovarian tumor tissue blocks were immediately rinsed with PBS and transported to the imaging laboratory within 60 minutes, stored in an ice box. The tissue slices were obtained by embedding and sectioning after being fixed in 4% formaldehyde for 24 hours at 4 ℃.

Post-fixation, samples underwent standard histological processing, including dehydration through a graded ethanol series (70%–100%), clearing in xylene, and infiltration with molten paraffin. The tissues were subsequently embedded in paraffin blocks. Serial sections of 5 μm thickness were cut using a rotary microtome and mounted on glass slides.

Note S2. Imaging system and image acquisition

TPAF imaging was performed using a commercial two-photon fluorescence microscope (FVMPE-RS, Olympus) with a water-immersion objective (XLUMPLFLN20XW, 20×, NA 1.0). After excitation using a tunable Ti:Sapphire femtosecond laser (Chameleon Ultra II, Coherent), the excited fluorescence signals were collected using two non-descanned photomultiplier tubes (PMTs). The excitation wavelength was set to 860 nm to simultaneously excite NADH and FAD. Two bandpass filters of BA575-645 and BA495-540 were inserted into FAD and NADH channels, respectively. Please refer to Supplementary Information for detailed description of TPAF imaging setup (Fig. S7).

TPAF images (1024×1024 pixels) were acquired with a field of view (FOV) of 636.396×636.396 µm (0.621 µm/pixel). The pixel dwell time was 2.0 µs. To characterize the noise statistics of the imaging system, A total of 51 FOVs was recorded, and for each FOV, TPAF images was acquired under four frame-averaging conditions (1×, 4×, 8×, 16×).

The corresponding H&E-stained slides, including tissue micro arrays (TMA) and whole slide images (WSI), were digitized using a commercial Hamamatsu NanoZoomer whole-slide scanner with a pixel resolution of approximately 0.50 µm/pixel. The images were saved in pyramidal SVS format to facilitate multi-scale navigation, and converted to pyramidal TIF format with a custom script for processing and analysis.

Note S3. Denoising dataset generation.

Training the denoising network requires pairs of clean I_{clean} and noisy I_{noisy} images. Since noise-free images in dynamic tissue are unattainable without complex image registration, we adopted a synthetic degradation strategy. We gathered a diverse corpus of high-SNR microscopy images from six public datasets to serve as noise-free references I_{clean}, i.e., the cytoplasmic fluorescence channels from these datasets as their textural characteristics (diffuse signal with negative nuclear contrast) closely approximate the TPAF appearance of ovarian stroma and epithelium:

Dataset	Description
BBBC007 (Drosophila Kc167 cells)	16 FOVs containing actin fluorescence (Broad Bioimage Benchmark Collection);
BBBC008 (HT29 Colon Cancer)	12 FOVs of cytoplasmic stains;
BBBC020 (Murine Macrophages)	25 FOVs containing CD11b/APC surface markers;
Cellpose	A subset of 118 images exhibiting diverse cellular morphologies;
MP6843	A dataset containing 100 FOVs of mouse neuroblastoma cells with filaments actin and cell skeletons stained with Phalloidin FITC;
NeurIPS 2022 Cell Segmentation Challenge	A multi-modality dataset containing 1000 labeled images and 1725 unlabeled images.

The six public datasets above collectively provide 1271 full-resolution source images. After randomly cropping to 512×512 patches and applying geometric augmentations, the effective training pool for denoising comprises ~1500 patches from public datasets and ~1000 patches from in-house dataset. For each patch on each training step, one of the three empirical noise levels (n = 1, 4, 8 frames) is randomly sampled and the corresponding synthetic noisy image is generated on-the-fly using the procedure described below.

Mixed Poisson-Gaussian model. Following standard practice for PMT-based fluorescence microscopy, we model the per-pixel noise as a mixed Poisson-Gaussian (MPG) process accounting for shot noise from quantized photon emission/detection and additive Gaussian readout noise from the PMT and digitization electronics. We emphasize that this MPG functional form is a generic detector model and does not by itself encode two-photon excitation physics (e.g., two-photon absorption cross-sections, point-spread function); its role here is to faithfully reproduce the noise statistics of our specific imaging system at the photon-starvation regime, and the parameters (g, σ_read) are calibrated empirically from multi-frame TPAF acquisitions on our system.

The 16-frame-averaged image I_{16} is taken as the noise-free reference, and the 1-, 4-, 8-frame-averaged images (I_1, I_4, I_8) are treated as noisy samples at three empirical noise levels. For an n-frame acquisition the noise model is:

$$I_{f_n} = \frac{1}{g} Poisson(g \cdot I_{16}) + N(0, \sigma_{read}^2)$$

where g denotes the effective detector gain (photons-to-digital-units conversion) and σ_{read}^2 is the variance of additive Gaussian readout noise.

Per-frame-level parameter calibration. For each empirical noise level n ∈ {1, 4, 8}, the calibration procedure is:

1. Compute the pixelwise difference image: $D\{f_n\} = I\{f_n\} - I_{16}$.
2. Partition each FOV into non-overlapping patches of size P × P pixels (P=32); within each patch, compute the local mean μ of I_{16} and the sample variance v of D_{f_n}, where
$$v = \frac{1}{N-1} \sum_{i,j} (D_{f_n}(i,j) - \overline{D_{f_n}})^2$$
with $N = P^2$ the number of pixels in the patch.
3. Aggregating (μ, v) pairs across all patches in an FOV and across all 51 FOVs yields a scatter of points to which we fit the variance-mean relation expected for an MPG process:
$$v = \frac{1}{g}\mu + \sigma_{read}^2$$
giving an estimate of g and σ_{read}^2 at noise level n. The resulting noise-parameter tuples $\theta_n = (g_n, \sigma_{read,n}^2)$ for n ∈ {1, 4, 8} are reported in Supplementary Table S4.

Frame-dependence of ($\boldsymbol{g}, \boldsymbol{\sigma_{read}}$). The calibrated parameters depend on n because frame averaging modifies both shot noise and readout noise. The readout-noise term follows the expected scaling $\boldsymbol{\sigma_{read,n}} \approx \frac{\boldsymbol{\sigma_{read,1}}}{\sqrt{\boldsymbol{n}}}$ (verified to within ±3% across n = 1, 4, 8; Table S4), consistent with averaging n independent, uncorrelated readout-noise realizations. The gain term g_n increases monotonically with n but sub-linearly relative to the ideal $g_n = n \cdot g_1$ (Table S4), which we attribute to the 8-bit quantization of the digitized intensities in the low photon-count regime. Modelling each n-frame-averaged image as a single MPG draw with the empirically fitted parameters ($g_n, \sigma_{read,n}$) therefore reproduces the measured noise statistics at each level.

Synthesis of training noise. For each clean reference I_{16}, a synthetic noisy image at randomly chosen noise level n is generated as:

$$I_{synthetic,n} = \frac{1}{g_n} Poisson(g_n \cdot I_{16}) + N(0, \sigma_{read,n}^2)$$

where n is sampled uniformly from {1, 4, 8} (corresponding to the three calibrated noise levels) at each training step.

Sensitivity of AFN-DeSeg to noise-parameter mismatch. To assess the robustness of AFN-DeSeg under deployment to a different optical platform, where (g, σ_{read}) will not match the calibrated values, we performed a sensitivity analysis in which we deliberately perturbed the calibrated parameters by ±10%, ±25%, and ±50%, regenerated synthetic noisy test data with the perturbed parameters, and evaluated the pre-trained AFN-DeSeg model (no retraining) on each mismatched test set. PSNR, SSIM, Dice, and mAP were computed at every perturbation level on the same N = 500 test FOVs. The results are summarized in Supplementary Table S1; the implications for cross-system generalization are discussed in the Discussion (Limitation I).

Note S4. Description and training details of H&E image pathology analysis models.
To generate high-quality ground truth labels and perform comparative analysis on Hematoxylin and Eosin (H&E) stained slides, we utilized two state-of-the-art computational pathology models: CLAM for slide-level subtyping and key diagnostic areas identification, and HoVer-Net for precise nuclei segmentation in H&E WSI and TMA images, serving as a computational reference standard.

S4.1 CLAM (Clustering-constrained Attention Multiple instance learning)
Method Description
CLAM is a high-throughput, weakly-supervised deep learning framework designed for Whole Slide Image (WSI) classification. Unlike fully supervised methods that require pixel-level annotations, CLAM operates under the Multiple Instance Learning (MIL) paradigm. It aggregates patch-level features extracted from the WSI to make a slide-level prediction. The core innovation of CLAM lies in its attention-based pooling mechanism, which assigns an attention score to each patch, indicating its diagnostic relevance. Furthermore, it employs instance-level clustering to constrain the feature space, ensuring that highly attended patches (evidence of malignancy) are linearly separable from non-attended patches. In this study, we utilized CLAM to identify diagnostic key regions and to classify ovarian cancer subtypes from H&E slides.
Dataset: UBC-OCEAN
The model was trained and evaluated using the UBC-OCEAN (University of British Columbia - Ovarian Cancer Subtype Classification and Outlier Detection) dataset. This is a large-scale, public histopathology dataset specifically curated for ovarian carcinoma subtyping. The dataset comprises over 500 whole slide images derived from ovarian cancer tissue samples. It covers the five major histotypes of ovarian carcinoma: High-Grade Serous Carcinoma (HGSC), Low-Grade Serous Carcinoma (LGSC), Endometrioid Carcinoma (EC), Clear Cell Carcinoma (CCC), and Mucinous Carcinoma (MC). The diversity and scale of this dataset ensure that the CLAM model learns robust feature representations specific to ovarian tissue morphology, making it highly suitable for generating attention heatmaps for our downstream validation.
We strictly followed the training and testing protocols described in the original CLAM publication and the official GitHub repository. Tissue regions were automatically segmented from the background, and non-overlapping patches of 256*256 pixels were extracted at 20x magnification. A UNI pathology foundation model, pre-trained on H&E images, was used to extract a 1024-dimensional feature vector for each patch. These features served as the input for the CLAM network. The model was trained using the Adam optimizer with a learning rate of $2*10^{-4}$ and a weight decay of $1*10^{-5}$. We employed the standard split for 10-fold cross-validation. For our analysis, we extracted the attention maps generated by the trained model to visualize and quantify the "key diagnostic areas", which served as a reference for evaluating the clinical relevance of our key diagnostic area predictions by TPAF nuclear masks.

S4.2 HoVer-Net (Horizontal-Vertical Net)
HoVer-Net is a segmentation and classification network designed for processing H&E images. It addresses the challenge of separating touching or overlapping nuclei, which is common in dense cancer tissues. The architecture consists of a shared encoder and three distinct decoder branches:

1. Nuclear Pixel Prediction: Predicts whether a pixel belongs to a nucleus.
2. HoVer Map Prediction: Predicts the horizontal and vertical distances of each nuclear pixel to its center of mass. The gradients of these maps are used to effectively separate clustered nuclei.
3. Nuclear Classification: Predicts the type of each segmented nucleus.

We utilized the pre-trained weights of HoVer-Net, which were trained on the PanNuke (Pan-Cancer Nuclei Segmentations) dataset.
In this study, HoVer-Net was used in inference mode to generate ground truth nuclei masks for corresponding H&E images. We directly loaded the model weights pre-trained on the PanNuke dataset. No fine-tuning was performed to maintain a standard baseline. Input H&E images were processed using the standard inference pipeline. Images were tiled with overlap to prevent edge artifacts. The post-processing steps, including the energy landscape watershed algorithm used to define nuclear boundaries based on HoVer maps, followed the default parameters specified in the HoVer-Net documentation.

Note S5. Description and training details of benchmark methods.

To assess the performance of our proposed AFN-DeSeg framework under fair and strict conditions, we evaluated it against six representative methods. These benchmarks collectively cover classical non-learning algorithms, supervised deep learning restoration, self-supervised blind denoising, and joint denoising-segmentation frameworks. For all learning-based methods, training and validation were performed using the exact same data splits defined in **Methods Section 4.1** to ensure comparability.

S5.1 BM3D (Block-Matching and 3D Filtering)

BM3D is a classical non-learning denoising algorithm widely accepted as a gold standard for removing additive Gaussian noise. While TPAF noise is signal-dependent (Poisson-Gaussian mixture), BM3D remains a robust baseline. In our evaluation, we applied the standard BM3D algorithm directly to each noisy TPAF test image. The noise standard deviation (σ) was estimated automatically from the input noisy image using the algorithm's built-in noise estimator (PSD-based). Hard-thresholding stage: Patch size of 8*8 pixels with a search window of 39*39 pixels. Wiener filtering stage: Patch size of 8*8 pixels with a search window of 39*39 pixels.

S5.2 CARE (Content-Aware Image Restoration)

We implemented the CARE framework using the canonical CSBDeep configuration. The network architecture is a standard U-Net with a depth of 4. The encoder consists of 4 down-sampling blocks. Each block comprises two convolutional layers with 3*3 kernels, followed by Batch Normalization and ReLU activation. Down-sampling is performed via 2*2 max-pooling. The number of feature channels doubles at each depth, starting from 32 filters in the first layer. The decoder path is symmetric, utilizing bilinear up-sampling. The model was trained in a supervised manner mapping noisy input images to clean reference targets. We utilized the Adam optimizer with an initial learning rate of $1*10^{-4}$ and a weight decay of $1*10^{-5}$. Training ran for 120 epochs with a batch size of 8 or 16. We employed a ReduceLROnPlateau scheduler, reducing the learning rate by a factor of 0.5 if the validation loss did not improve for 10 epochs. The optimization objective was the pixel-wise mean absolute error (MAE / L_1 loss) between the network prediction and the clean ground truth. The model checkpoint with the lowest validation L_1 loss was selected for testing.

S5.3 Noise2Fast (N2F)

To evaluate a self-supervised / blind denoising baseline where no clean ground truth is available, we employed Noise2Fast (N2F). We utilized a U-Net backbone similar to the original N2F/Noise2Void specification. The network was trained exclusively on the noisy images from the training split. The model utilized a blind-spot masking scheme where the center pixel of the receptive field is masked out during training, forcing the network to predict the pixel value based on its local neighborhood. We trained the model for 50 epochs using the Adam optimizer with a learning rate of $1*10^{-3}$ and a batch size of 16. The input images were cropped into patches of 64*64 pixels with random rotations and flips for data augmentation. During inference, the full image was processed. Since this is a self-supervised method, no clean reference data was used during the training phase.

S5.4 DenoiSeg Model

We implemented DenoiSeg using its standard published configuration designed for joint denoising and segmentation. The model features a shared U-Net encoder that feeds into two separate decoder heads: one for denoising (outputting intensity maps) and one for segmentation (outputting three classes: background, foreground, and border). The model was trained on the annotated portion of our dataset (noisy input images paired with clean images and human-corrected nuclei masks). The total loss L_{total} is a weighted sum of the denoising and segmentation losses:

$$L_{total} = \lambda_{rec} L_{rec} + \lambda_{seg} L_{seg}$$

where L_{rec} is the L1 reconstruction loss and L_{seg} is the sum of Dice loss and binary cross-entropy (BCE) loss. We set the weights to λ_{rec} = 1.0 and λ_{seg} = 2.0 based on the original paper's recommendation for high-noise regimes. Training employed the Adam optimizer with a learning rate of $5*10^{-4}$ and weight decay of $1*10^{-5}$ for a batch size of 4. The maximum epochs were set to 100, with an early stopping mechanism triggered if the validation segmentation Dice score did not improve for 10 consecutive epochs. The best checkpoint was selected based on the highest validation Dice score.

S5.5 Cellpose 3 Model

We re-trained the Cellpose 3 model to serve as a strong supervised segmentation benchmark. We utilized the standard Cellpose architecture with the "nuclei" style backbone. This architecture predicts horizontal and vertical spatial flow gradients along with a cell probability map. Input images were resized such that the average nuclear diameter approximated 30 pixels, adhering to the recommended Cellpose scaling convention. The model was trained from scratch using our training set of noisy images and corresponding ground-truth masks. We used the AdamW optimizer with an initial learning rate of $1*10^{-4}$ and weight decay of $1*10^{-5}$. Training proceeded for 100 epochs with a batch size of 4. A cosine annealing learning rate scheduler was applied. Model checkpoints were saved every 10 epochs, and the final model was selected based on the highest validation Dice coefficient.

S5.6 ResNet50 Model

To disentangle the contribution of the DINOv3-based dual-encoder design from the more general benefit of using any pre-trained backbone, we trained an additional ResNet50-UNet baseline. The encoder is an ImageNet-pre-trained ResNet-50 (torchvision. models. resnet50 weights), modified to accept the same single-channel TPAF input with the same ImageNet normalization as AFN-DeSeg. Multi-scale features at the four standard ResNet stages (output strides 4, 8, 16, 32) are passed via skip connections to a symmetric U-Net-style decoder consisting of four bilinear-upsampling blocks, each comprising 3×3 convolution, BatchNorm, ReLU, 3×3 convolution, BatchNorm, ReLU. The decoder branches into two parallel heads: a denoising head outputting a single-channel intensity map (1×1 convolution) and a segmentation head outputting a pixel-wise probability map (1×1 convolution + sigmoid), mirroring the AFN-DeSeg dual-decoder structure. The total trainable parameter count is 32.5M, comparable to AFN-DeSeg within a factor of two. Training used the same data splits, the same instrument-calibrated MPG noise synthesis (Note S3), and the identical joint loss ($\lambda\_rec = 1.0$, $\lambda\_seg = 5.0$, $\lambda\_percep = 0.1$) as AFN-DeSeg. The optimizer was AdamW (weight decay 0.05, gradient clipping max-norm 1.0), with an initial learning rate of 1×10^{-4} following a cosine annealing schedule down to 1×10^{-6}, batch size 4, trained for 150 epochs with the same early-stopping criterion (segmentation Dice on validation, patience 20 epochs) as AFN-DeSeg. Geometric augmentations were also identical. This setup isolates the encoder family (ResNet50 with ImageNet pre-training, single stream) as the sole variable distinguishing this baseline from AFN-DeSeg's DINOv3 dual-stream design.

S5.7 Baseline Segmentation Model for Denoising-Only Methods

Since classical (BM3D) and denoising (CARE, Noise2Fast) methods only generate denoised intensity images, a separate downstream segmentation step is required to evaluate their diagnostic utility. To ensure a fair comparison, we trained a standardized **fine-tuned Cellpose cyto2 model** to act as the universal segmenter for these methods. This establishes a "Denoise-then-Segment" pipeline, allowing us to quantify how much the upstream image quality improvement contributes to downstream segmentation accuracy. The generic Cellpose cyto2 model (pre-trained on Cellpose microscopy dataset) was fine-tuned on our TPAF training dataset. Specifically, it was trained to map original TPAF images to human-corrected ground truth masks with a learning rate of $1*10^{-4}$ and a batch size of 8 for 100 epochs. During testing, the denoised images generated by BM3D, CARE, and Noise2Fast were fed into this fixed segmentation model without any further re-training. This isolates the restoration quality as the sole variable affecting segmentation performance differences.

Note S6. Definition of evaluation metrics.
To rigorously quantify the performance of the AFN-DeSeg framework, we employed a comprehensive set of metrics spanning signal fidelity, algorithmic segmentation accuracy, biological morphology preservation, and clinical diagnostic utility.

S6.1 Image Restoration Metrics

These metrics quantify the fidelity of the denoised TPAF images (I_{pred}) compared to the clean/reference images (I_{ref}).

1. Peak Signal-to-Noise Ratio (PSNR)

PSNR measures the ratio between the maximum possible power of a signal and the power of corrupting noise, expressed in decibels (dB).

$$PSNR = 10 \cdot log_{10}(\frac{MAX_I^2}{MSE})$$

Where MAX_I is the maximum possible pixel value of the image (e.g., 255 for 8-bit images or 65535 for 16-bit images). MSE is the Mean Squared Error defined as:

$$MSE = \frac{1}{mn}\sum_{i=0}^{m-1}\sum_{j=0}^{n-1}[I_{ref}(i,j) - I_{pred}(i,j)]^2$$

where m and n are the image dimensions.

2. Structural Similarity Index (SSIM)

SSIM evaluates the perceived quality of digital images by analyzing the inter-dependencies between pixels, focusing on luminance, contrast, and structure.

$$SSIM(x,y) = \frac{(2\mu_x\mu_y + C_1)(2\sigma_{xy} + C_2)}{(\mu_x^2 + \mu_y^2 + C_1)(\sigma_x^2 + \sigma_y^2 + C_2)}$$

where μ_x, μ_y are the local means, σ_x^2, σ_y^2 are the local variances, and σ_{xy} is the covariance of images x (reference) and y (denoised). C_1 and C_2 are small constants to stabilize the division.

S6.2 Segmentation Metrics

These metrics evaluate the accuracy of nuclear detection and segmentation masks (M_{pred}) against human-corrected ground truth masks (M_{gt}).

1. Dice Coefficient (DC)

DC measures the spatial overlap accuracy between the predicted binary mask and the ground truth.

$$DC = \frac{2|M_{pred} \cap M_{gt}|}{|M_{pred}| + |M_{gt}|} = \frac{2 \cdot TP}{2 \cdot TP + FP + FN}$$

where TP (True Positive), FP (False Positive), and FN (False Negative) are pixel-level counts.

2. Mean Average Precision (mAP @ IoU 0.5)

The metric mAP evaluates instance-level detection performance. We first define the IoU for a predicted object instance A and a ground truth object B:

$$IoU = \frac{A \cap B}{A \cup B}$$

A detection is considered a True Positive if IoU > 0.5. The Average Precision (AP) is the area under the Precision-Recall curve. The mAP is the mean AP calculated across all images in the test set.

S6.3 Nuclei Morphological and Statistical Evaluation Metrics

To ensure pathological relevance, we quantified morphological features, including nuclear area, nuclear circularity, and nuclear density.

1. Nuclear Area (A_{nuc})

The total physical size of a nucleus is defined as the pixel count N_{pix}.

$$A_{nuc} = N_{pix}$$

The unit can be converted to physical units like μm^2 if the image resolution is specified.

2. Nuclear Circularity (C_{nuc})

Nuclear atypia is an important morphological biomarker for cancer diagnosis, so nuclear circularity is used to quantify atypia. A value of 1.0 indicates a perfect circle, while values approaching 0 indicate highly irregular shapes.

$$C_{nuc} = \frac{4\pi \cdot A_{nuc}}{(P_{nuc})^2}$$

where P_{nuc} is the perimeter of the nucleus boundary (in number of pixels).

3. Nuclear Density (D_{FOV})

A measure of tumor cellularity, defined as the number of segmented nuclei (N_{total}) per unit tissue area A_{FOV} within an FOV.

$$D_{FOV} = \frac{N_{total}}{A_{FOV}}$$

S6.4 Key Diagnostic Area Prediction Validation Metrics

To validate the clinical application potential of AFN-DeSeg, we assessed the quantitative equivalence between TPAF-derived nuclear map and diagnostic predictions and those of standard H&E histopathology. The key diagnostic area predictions are generated by the attention U-Net prediction model, which takes binary nuclei masks (from either TPAF or H&E images) as input to predict regions of high tumor burden.

1. Pearson's Correlation Coefficient (r)

To demonstrate the pathology equivalence, we quantified the linear relationship between TPAF nuclear prediction and manual H&E-based ground truth across 200 matched FOVs. The two key variables are nuclear density and key area fraction (KAF). The Pearson's Correlation Coefficient (r) is calculated for the two variables:

$$r = \frac{\sum(X_i - \bar{X})(Y_i - \bar{Y})}{\sqrt{\sum(X_i - \bar{X})^2 \sum(Y_i - \bar{Y})^2}}$$

Where X_i and Y_i respectively represent the variables (nuclear density or KAF) predicted from TPAF and H&E nuclear masks of the ith sample. $\bar{X}$ and $\bar{Y}$ represent their mean values of the test dataset.

2. Key Area Fraction (KAF)

The proportion of the tissue area classified as diagnostically relevant (high-attention) by the prediction model.

$$KAF = \frac{Area\ of\ Key\ Diagnostics\ Region\ Predicted}{Total\ Tissue\ Area}$$

3. Bland-Altman Analysis

To detect potential systematic errors and evaluate the reliability of AFN-DeSeg as an assistive tool, we performed Bland-Altman analysis on KAF results of key diagnostic regions predicted by TPAF (X_i) and H&E (Y_i) nuclear masks.

Bias ($\bar{d}$)**:** The mean difference between methods:

$$\bar{d} = \frac{1}{n}\sum_{i=1}^{n}(X_i - Y_i)$$

Limits of Agreement (LoA): Defined as the interval containing 95% of the differences:

$$LoA = \bar{d} \pm 1.96 \cdot SD_{diff}$$

where SD_{diff} is the standard deviation of the differences.

4. 95% Hausdorff Distance (HD95)

HD95 measures the precision of surgical boundaries by calculating the 95th percentile of the minimum Euclidean distances between points on the predicted boundary (P) and the ground truth boundary labeled by CLAM model and pathologists (G).

$$HD95(P, G) = \max(h_{95}(P, G), h_{95}(G, P))$$

where $h_{95}(P, G)$ is the 95th percentile of the set of distances $\{min_{g\in G}\|p - g\| \mid p \in P\}$.

Table S1. Sensitivity of AFN-DeSeg performance to noise-model parameter mismatch.
The instrument-calibrated noise parameters (g, σ_{read}) of the Olympus FVMPE-RS system (baseline values from Note S3 / Table S4) were perturbed by ±10%, ±25%, and ±50%; synthetic noisy test data were regenerated using the perturbed parameters, and the pre-trained AFN-DeSeg model was evaluated on each perturbed test set. Values are mean ± std across N = 500 test FOVs. ΔPSNR / ΔSSIM / ΔDice / ΔmAP columns report the change relative to the baseline (0% perturbation).

Perturbation	PSNR (dB)	ΔPSNR	SSIM	ΔSSIM	Dice	ΔDice	mAP@IoU=0.5	ΔmAP
0% (baseline)	28.5 ± 2.5	0.0	0.75 ± 0.06	0.00	0.80 ± 0.10	0.00	0.70 ± 0.11	0.00
+10%	28.4 ± 2.5	-0.1	0.75 ± 0.06	0.00	0.80 ± 0.10	0.00	0.69 ± 0.11	-0.01
−10%	28.1 ± 2.6	-0.4	0.74 ± 0.06	-0.01	0.79 ± 0.11	-0.01	0.68 ± 0.12	-0.02
+25%	28.0 ± 2.6	-0.5	0.74 ± 0.06	-0.01	0.79 ± 0.11	-0.01	0.68 ± 0.11	-0.02
−25%	27.2 ± 2.7	-1.3	0.72 ± 0.07	-0.03	0.76 ± 0.12	-0.04	0.65 ± 0.12	-0.05
+50%	26.5 ± 2.5	-2.0	0.68 ± 0.06	-0.07	0.71 ± 0.11	-0.09	0.60 ± 0.14	-0.10
−50%	25.7 ± 2.8	-2.8	0.66 ± 0.08	-0.09	0.68 ± 0.13	-0.12	0.56 ± 0.13	-0.14

Footnote: Same N = 500 test set as Figures 3c/d and 4b/c. Both g and σ_{read} are perturbed by the same fractional amount in each row (joint perturbation); independent perturbation of each parameter yields qualitatively similar results (data not shown). Evaluated at the single-frame (n = 1) noise level.

Table S2. Annotation pipeline statistics for the in-house TPAF segmentation dataset.

**Metric**	**Value**
Total number of annotated FOVs	~300 (512*512 px)
Total annotated nuclei instances	~130,000
Number of annotators	1
Mean Dice (raw fine-tuned cyto2 vs. final corrected mask)	0.72
Edits - fraction adding missed nuclei in low-contrast regions	38.5%
Edits - fraction removing false positives in cytoplasmic patches/vesicles	16.2%
Edits - fraction reshaping boundaries of irregular nuclei	32.6%
Pathologist-flagged FOVs requiring second-round correction	13.4%
Average annotation time per FOV	~20 min
Total human annotation effort	~100 hours

Table S3. Full numerical comparison of AFN-DeSeg against all benchmark methods at three empirical noise levels (n = 1, 4, 8 frames). All learning-based methods shared the identical training, validation, and test splits as AFN-DeSeg; BM3D is non-learning. Values are mean ± std across N = 500 test FOVs.

Method	Pre-training	Noise level	PSNR (dB)	SSIM	Dice	mAP@IoU=0.5
**BM3D**	none	n=1 (high noise)	19.6 ± 2.7	0.33 ± 0.07	0.47 ± 0.14	0.27 ± 0.13
		n=4 (mid noise)	26.4 ± 1.9	0.55 ± 0.05	0.59 ± 0.09	0.47 ± 0.11
		n=8 (low noise)	30.7 ± 1.3	0.71 ± 0.04	0.67 ± 0.07	0.61 ± 0.08
**CARE**	none	n=1	24.3 ± 2.7	0.59 ± 0.06	0.63 ± 0.12	0.47 ± 0.11
		n=4	30.1 ± 1.8	0.72 ± 0.04	0.74 ± 0.07	0.63 ± 0.08
		n=8	35.6 ± 1.5	0.81 ± 0.02	0.79 ± 0.05	0.75 ± 0.06
**Noise2Fast**	none	n=1	23.7 ± 3.1	0.47 ± 0.08	0.57 ± 0.11	0.40 ± 0.12
		n=4	28.3 ± 2.5	0.66 ± 0.06	0.69 ± 0.08	0.58 ± 0.09
		n=8	33.8 ± 2.1	0.75 ± 0.05	0.75 ± 0.06	0.70 ± 0.07
**DenoiSeg**	none	n=1	23.9 ± 2.8	0.49 ± 0.07	0.54 ± 0.12	0.39 ± 0.12
		n=4	28.6 ± 2.3	0.71 ± 0.06	0.64 ± 0.09	0.54 ± 0.11
		n=8	33.2 ± 1.7	0.78 ± 0.04	0.71 ± 0.07	0.66 ± 0.09
**Cellpose 3**	none	n=1	24.1 ± 2.6	0.55 ± 0.07	0.69 ± 0.09	0.56 ± 0.09
		n=4	27.9 ± 1.8	0.76 ± 0.04	0.77 ± 0.08	0.70 ± 0.07
		n=8	32.4 ± 1.6	0.83 ± 0.03	0.83 ± 0.05	0.79 ± 0.05
**ResNet50**	ImageNet	n=1	26.1 ± 2.4	0.63 ± 0.06	0.74 ± 0.11	0.55 ± 0.10
		n=4	30.6 ± 1.9	0.79 ± 0.03	0.81 ± 0.06	0.70 ± 0.08
		n=8	35.4 ± 1.4	0.86 ± 0.02	0.85 ± 0.04	0.81 ± 0.06
**AFN-DeSeg**	DINOv3+LoRA	n=1	28.5 ± 2.5	0.75 ± 0.06	0.80 ± 0.10	0.70 ± 0.11
		n=4	33.1 ± 1.6	0.83 ± 0.04	0.85 ± 0.07	0.79 ± 0.07
		n=8	37.9 ± 1.2	0.89 ± 0.03	0.88 ± 0.05	0.86 ± 0.05

Footnote: BM3D, CARE, and Noise2Fast are denoising-only methods; their downstream Dice and mAP were computed by pairing the denoised outputs with the fine-tuned cyto2 segmenter described in Note S5.7, establishing a standardized "Denoise-then-Segment" pipeline. This allows direct comparison of upstream denoising quality on the same downstream segmentation backbone. Cellpose 3, DenoiSeg, ResNet50-UNet, and AFN-DeSeg perform end-to-end joint denoising and segmentation. ResNet50-UNet shares the identical training protocol (joint loss, optimizer, schedule, data splits, and instrument-calibrated MPG noise synthesis) as AFN-DeSeg; the only difference is the encoder architecture, introduced to disentangle the contribution of pre-trained representations from that of the proposed dual-encoder + LoRA design. The AFN-DeSeg Dice value of 0.88 at n=8 is obtained on a held-out test set whose patient identifiers are disjoint from the training set (patient-level split; see Note S1), confirming that this value reflects genuine generalization rather than data leakage.

Table S4. Calibrated noise-model parameters of the Olympus FVMPE-RS imaging system. Parameters are fitted per empirical noise level (n = 1, 4, 8 frames averaged) by the variance-mean fitting procedure described in Note S3. Values are mean ± std across the 51 calibration FOVs.

Noise level	Frames averaged (n)	Gain term g (8-bit DU)[1]	Readout noise std σ_{read} (8-bit DU)
**n=1 (high noise)**	1	g_1 = 0.038 ± 0.013	σ_read,1 = 13.06 ± 4.68
**n=4 (mid noise)**	4	g_4 = 0.116 ± 0.035	σ_read,4 = 6.37 ± 2.36
**n=8 (low noise)**	8	g_8 = 0.224 ± 0.066	σ_read,8 = 4.49 ± 1.70
**16 (reference)**	16	(used as I_{16} noise-free reference)	—

[1] g defined per Note S3 variance-mean relation $v = \frac{1}{g} \cdot \mu + \sigma_{read}^2$; values in 8-bit digital units (DU, 0-255 intensity scale), patch size 32×32, mean ± std across 51 calibration FOVs.

Footnote: DU = digital unit (PMT-digitized intensity). The dependence of $(g_n, \sigma_{read,n})$ on n arises from frame averaging: averaging n independent frames raises the effective accumulated photon count per DU (g_n increases sub-linearly with n) and reduces uncorrelated readout noise ($\sigma_{read,n} \approx \frac{1}{\sqrt{n}}$); see Note S3 for derivation.

Table S5. Consolidated dataset summary across all study purposes.
Sample counts at the patient, slide/sample, and FOV/patch levels are provided to clarify the biological independence and scale of each evaluation.

Purpose	Source	# Patients	# Sections	# FOVs / Patches
**Noise-model calibration**	In-house TPAF, cohort iii	4	4 TMA	51 multi-frame FOVs; used only to fit (g, $\sigma_{\_read}$); not in any train/val/test
**Joint Denoising and Segmentation Training**	6 public datasets	n/a	n/a	~1500 patches
	In-house TPAF, cohort ii	2	2 WSI	~1000 patches (from ~100 annotated FOVs of 2 patients); 10% held out as validation for early stopping
**Joint Denoising and Segmentation Test (Fig 3-4)**	In-house TPAF, cohort ii held-out	4	4 WSI	N = 500 (256×256 patches, 159×159 μm², from the ~200 held-out FOVs)
**Joint Denoising and Segmentation Test (Fig 5)**	Matched in-house TPAF + H&E, cohort ii held-out	4	4 WSI	N = 200 (512×512 FOVs, 318×318 μm²)
**Clinical key-area prediction (Fig. 6)**	TMA cohort (i) + WSIs of cohort (ii); H&E + labels	74	130 TMAs + 6 WSIs	Train: 30 TMA cores + 2 WSIs<br>Test: 100 TMA cores + 4 WSIs

Footnote**:** The three patient cohorts (i) TMA (68), (ii) WSI (6), (iii) calibration (4) are mutually exclusive at the patient level (78 patients total). The calibration cohort (iii) is used solely for MPG noise-model fitting (Table S4) and enters no training/validation/test set.
